# Neuropharmacology

**Published:** 2008-10

**264**

**Estrogen therapy improves neuronal cytoarchitecture, apoptotic index and estrogen receptor regulation in hormone depleted adult and in aged rat hippocampus**

Mehra R

All India Institute of Medical Sciences, New Delhi, India.

To understand the role of estrogen in aged rat hippocampus, we studied the effects of estrogen (E2) deprivation (with ovariectomy or by natural aging) and E2 (17-beta estradiol) replenishment therapy (a daily s.c. injection of E2, 0.1 mg/kg body wt. in 0.1 ml sesame oil for a period of 4 weeks) on estrogen receptor (ER) α and β regulation, neuronal cytoarchitecture and apoptosis. 30 adult female wistar rats (age=4-5 months) were included in this study. The animals were divided into 6 groups (n=5 each) consisting of Group A; ovary intact adult rats, Group B; ovariectomized (ovx) adult rats, Group C; E2 treated ovx adult rats, Group D; ovary intact aged rats, Group E; ovx aged rats, and Group F; E2 treated ovx aged rats (animals of Group D, E and F were kept for aging until 18 months). Analysis of results showed that the ovariectomy resulted in deterioration of hippocampal cytoarchitecture and increased apoptosis and the ER alpha and beta immunoreactive (ir) neurons were significantly (*P*<0.05) reduced in number when compared to the ovary intact adult controls. The E2 therapy proved to be beneficial and led to reversal of these changes. Hippocampus of ovary intact aged animals showed less deleterious effects when compared to age matched ovariectomized rats. E2 treated ovx aged rats showed improved neuronal cytoarchitecture, decreased apoptosis and increased number of ER alpha and beta immunoreactive neurons when compared to the age matched ovx control rats. Studies suggested that E2 maintains and regulates the hippocampal neuronal integrity. It could be beneficial in preventing or delaying the E2 deficit effects or the neurodegenerative aging process arising as a result of surgical or natural estropause, if given on a long term therapeutic basis.

**265**

**Mitochondria as therapeutic target of estrogen mediated neuroprotection**

Kumar Pavan, Varshney Mukesh K, Mehra Raj

All India Institute of Medical Sciences, New Delhi, India.

In continuation of our efforts to elucidate the role of estrogen in neuroprotection of hippocampal neurons, we studied the F1- ATPase (mitochondrial ATPase), Bcl-2 (anti-apoptotic protein) and Bax (pro-apoptotic protein) levels in estrogen deprived and estrogen replenished adult female rat hippocampus (using our ovariectomized rat animal model). 30 adult female wistar rats (age= 4-5 months) were included in this study and divided into three groups (n= 10 each) as follows: group1; ovary intact rats, group 2; ovariectomized (ovx) rats and group 3; 17β-estradiol (E2) treated ovx rats (a daily s.c. injection of E2, 0.1 mg/kg body wt. in 0.1 ml sesame oil for 4 weeks). Ovariectomy resulted in marked upregulation of Bax and F1-ATPase activity in various hippocampal subfields as compared to ovary intact rat hippocampus. On the other hand Bcl-2 expression was downregulated in the same hippocampal subfields. E2 therapy to ovx rats resulted in reversal of expression profiles of F1-ATPase, Bax and Bcl-2 proteins. Estrogen treatment decreased the expression of F1-ATPase and Bax in the ovx rat hippocampus while Bcl2 levels were increased following E2 therapy as compared to ovx rats. Estrogen therapy brought the expression of these proteins more or less near to the expression levels of ovary intact rat hippocampus. Our results indicate that the estrogen may mediate hippocampal neuroprotection via the mitochondrial involvement of F1-ATPase activity by preserving the ATP levels and thereby regulating the pro-apoptotic and anti-apoptotic proteins.

**266**

**Therapeutic interventions of *Withania somnifera* in oxygen glucose deprivation-pc-12 cell model of cerebral stroke**

Singh G^1^, Siddiqui MA^1^, Kashyap MP^1^, Pant KK^2^, Gupta YK^3^, Pant AB^1^

^1^Indian Institute of Toxicology Research, Lucknow; ^2^CSM Medical University, Lucknow; ^3^All India Institute of Medical Sciences, New Delhi, India.

Oxidative stress mediated neuronal injury is well documented in ischemic cerebral stroke. The numbers of antioxidants have been looked at for their neuroprotective potential and *Withania somnifera* is one of them. However, the mechanisms involved in therapeutic intervention of *W. somnifera* in cerebral stroke are yet to explore. Thus, attempts were initiated to identify the points of therapeutic interventions of *W. somnifera* using oxygen-glucose deprivation (OGD)-PC-12 cell *in vitro* model of ischemic stroke. The model was created by exposing the PC-12 cells to OGD insult for 6 h following a re-oxygenation of 24 h under normoxia condition in glucose containing medium. Biologically safe concentrations (25, 50, 100 µg/ml) of *W. somnifera* identified through MTT and LDH assays were used. Three treatment schedules were designed viz., pretreatment group (treatment for 24 h prior to OGD insult), post treatment group (treatment for 24 h after OGD insult) and whole treatment group (treatment starting from 24 h prior to OGD insult and continued till the completion of re-oxygenation period). Parallel sets without OGD insult, OGD insult without treatment also run under identical conditions and served as basal and OGD controls respectively. Following respective treatments, cells were analyzed to study the *W. somnifera* mediated restoration of oxidative stress induced alterations viz., reactive oxygenation species (ROS), nitric oxide (NO), lipid peroxidation (LPO), glutathione content (GSH), membrane potential, dopamine receptor (DA-D_2_) and prostaglandin E_2_ (PGE_2_). In general, a dose dependent significant recovery in the levels of PGE_2_, GSH and NO could be recorded in all three treatment groups, however, pre- and post-treatment groups were found to be most effective on ROS and DA-D_2_ restoration. No significant response could be detected for LPO and membrane potential. The preliminary finding suggests the therapeutic intervention of *W. somnifera* in cerebral stroke by affecting a range of endpoints. However, further experiments are needed to reach any firm conclusion.

**267**

**Study to evaluate correlation between experimentally induced various inflammatory models seizure and biochemical parameters in rats**

Medhi B, Rao R S, Khanduja K L^1^, Pandhi P

Department of Pharmacology and Biophysics^1^, Postgraduate Institute of Medical Education and Research, Chandigarh, India.

**Background:**

Oxidative stress has been implicated in the pathogenesis of various conditions including epilepsy, inflammatory bowel disease and rheumatoid arthritis (RA). Aim of the study was to produce various inflammatory models and seizure and to understand the effect of different drugs (thalidomide, etoricoxib) on seizure and to find out the correlation with antioxidant parameters.

**Materials and Methods:**

Total of 54 male rats was included in the study. Rats were divided into 3 groups of acetic acid colitis, adjuvant arthritis, and cotton wool granuloma. Each group had 3 subgroups of control, model and treatment. Thalidomide was used as treatment in colitis and arthritis group while etoricoxib was used in cotton wool granuloma group. At the end of three days in colitis, seventeen days in arthritis and seven days in cotton wool granuloma group a subconvulsive dose of pentylenetetrazole (PTZ) (40mg/kg i.p.) was injected intraperitoneally to note seizure onset and seizure score. Presence of inflammation was confirmed by morphology and histology. Plasma and brain biochemical parameters like Malondialdehyde (MDA), Superoxide dismutase (SOD), Glutathione peroxidase (GPx) were estimated.

**Results:**

The models of colitis and arthritis were effectively produced as evidenced by morphology scores (*P*<0.001). Seizure onset was reduced and grade was increased (*P*<0.001). Thalidomide reduced the morphological (*P*<0.002) and seizure grade (*P*<0.001) while increased seizure onset (*P*<0.001) in the arthritis group. There was an increase in MDA levels in the brain of thalidomide treated arthritis group (*P*<0.05) while there was a no significant raise in SOD and GPx levels. Conclusion: Inflammation is associated with decreased threshold to PTZ induced seizure. Thalidomide is effective in reducing the extent of arthritis as well as reducing the seizure scoring and increasing seizure onset in the adjuvant arthritis group. As it increased lipid peroxidation and reduced SOD and GPx, further evaluation is necessary with respect to oxidative stress.

**Conclusion:**

Inflammation is associated with decreased threshold to PTZ induced seizure. Thalidomide is effective in reducing the extent of arthritis as well as reducing the seizure scoring and increasing seizure onset in the adjuvant arthritis group. As it increased lipid peroxidation and reduced SOD and GPx, further evaluation is necessary with respect to oxidative stress.

**268**

**Effect of anticonvulsant activity of some novel 2-substituted-1, 3-oxazolidines**

Mathur AS, Vachala SD, Chaturvedi P, Bhavna K, Chaubey N, Shilpee, Haris N

Manipal College of Pharmaceutical Sciences, Manipal University, Manipal, Karnataka, India.

**Introduction:**

Following the discovery of Oxazolidine nucleus, numerous of structural modifications have been made to increase their biological activities. In the present study, a new series of 3-(2- furyl methyl)-4-phenyl-2-substituted -1, 3-Oxazolidine derivatives were synthesised and studied for their anticonvulsant activity.

**Materials and Methods:**

1, 3-Oxazolidine derivatives were synthesised by condensation of reduced Schiff base of phenyl glycinol with different aromatic / hetero aromatic aldehydes. The chemical structures were conformed by means of IR, ^1^H-NMR, ^13^C-NMR and mass spectral analysis. The anticonvulsant activity was done by Maximal electro shock seizure method.

**Results:**

Compound 4-[3-(2- furylmethyl)-4-phenyl-1, 3-oxazolidin-2-yl]-2-methoxy phenol 11 and compound 4-[3-(2-furylmethyl)-4-phenyl-1, 3-oxazolidin-2-yl]phenol 12 exhibited the highest anticonvulsant activity by Maximal electro convulsio method. 3-(2-furylmethyl)-2-(3-nitro phenyl)-4-phenyl-1, 3-oxazolidine 8 and 3-[3-(2-furylmethyl) 4-phenyl-1, 3-oxazolidin- 2-yl]-1H-indole 4 showed moderate activity where as the remaining compounds showed very lesser activity when compared with the standard.

**Conclusions:**

From study, the data revealed that compound containing -OCH_3_, -NO_2_ and -OH groups substituted phenyl ring at Para and meta position was found to increase the biological activities. Therefore substitutions at 2^nd^ position in 1, 3-Oxazolidines seems to play a vital role in anticonvulsant activity.

**269**

**To compare the efficacy, safety and tolerability of olanzapine and sodium valproate given alone or as add on therapy in acute mania**

Gupta M, Jiloha, R C Tekur U, Kumar A

Maulana Azad Medical College, New Delhi, India.

Acute mania is a psychiatric disorder which requires hospitalisation and prompt control of symptoms. The standard drug treatment of acute mania follwed in our hospital is Haloperidol along with anticholinergics like Trihexiphenidyl and/or atypical antipsychotics like Olanzapine. The aim of present study was to compare safety, efficacy and tolerability of Sodium Valproate and Olanzapine administered alone or in combination in patients suffering from acute mania.

**Materials and Methods:**

A total of 30 patients suffering from acute mania based on DSM IV – TR criteria were divided into two equal groups. Group I was treated with Sodium Valproate 250mg thrice daily titrated to 20 mg/kg/day. Group II received Olanzapine 5 mg twice daily upto 20 mg/day. In both the groups sodium valproate or olanzapine were given as addon therapy at 3 weeks. The primary method of assessment was ≥ 50% improvement in YMRS (young mania rating scale) score on days 4, 7, 10, 14, 18 and 21. The serum levels of Valproic acid were also measured on days 4, 10, 14 and 21.

**Results:**

Both Sodium Valproate and Olanzapine were efficacious in the treatment of acute mania with all the patients showing a fall in YMRS scores by ≥ 50%at the end of the 21 day study period.. The difference in clinical response of two groups was not statistically significant (*P* < 0.05). However, Sodium Valproate treated patients who received Olanzapine in third week had 15.3% fall while patients on Olanzapine who received Sodium Valproate had a 23.7% fall in the YMRS score. Patients who attained serum valproic acid level of 100*µ*g/ml had an improvement in YMRS scores by≥ 50%.

**Conclusions:**

The present study reinforces the use of combination therapy in management of acute mania. The study also suggests that serum valproic acid levels of 100*µ*g/ml are necessary for clinical response.

**270**

**Augmentation of tonic gabaergic inhibition is associated with robust seizure control in chronic temporal lobe epilepsy in rats**

Briyal Seema, Reddy D Samba

Department of Neuroscience and Experimental Therapeutics, Texas AandM University Health Science Center, College of Medicine, College Station, Texas, USA.

Antiepileptic drugs are the mainstay for the treatment of epileptic seizures. Despite the availability of over 25 antiepileptic drugs, nearly 30% of people with epilepsy have intractable seizures that do not respond to even the best available treatment. Temporal lobe epilepsy (TLE) is a condition of progressive expansion of spontaneous recurrent seizures originating from the limbic system regions, specially the hippocampus. Augmentation of tonic GABAergic inhibition is an emerging strategy for designing novel antiepileptic drugs. In this study, we tested the hypothesis that selective augmentation of tonic GABAergic inhibition mediated by delta-subunit containing GABA-A receptors leads to effective inhibition of seizures in chronic epilepsy. To test this hypothesis, we utilized gaboxadol, which is a systemically active, delta-subunitpreferring GABA-A receptor agonist that enhances tonic GABAergic inhibition in the hippocampus. To test this novel strategy, we assessed the efficacy of gaboxadol in the rat pilocarpine model of TLE with spontaneous motor and electrographic seizures. The TLE was induced in rats by pilocarpine-induced status epilepticus and rats were monitored for spontaneous seizures during 2-6 months post pilocarpine. The efficacy of gaboxadol (5-10 mg/kg, sc) was evaluated in rats by monitoring frequency and duration of spontaneous and EEG seizures. Rats showed spontaneous seizures after latency of 60 days post pilocarpine. Gaboxadol therapy significantly decreased the frequency and duration of spontaneous seizures as well as EEG electrographic events. Pilocarpine model has several clinical features of human epilepsy. These key parameters were markedly reduced by gaboxadol treatment, confirming the efficacy of tonic GABAergic inhibition as an effective strategy for epilepsy therapy. ** Supported partly by NIH grant NS052158 **

**271**

**Effect of different doses of curcumin in phenobarbital resistant mice**

Chauhan A, Katyal J, Gupta YK

Department of Pharmacology, All India Institute of Medical Sciences, New Delhi, India.

**Introduction** and **Objective:**

Resistant epilepsy accounts for about 30 - 40% of epilepsy cases. As many side effects are associated with current antiepileptic drugs the use of herbal drugs proves lucrative. Therefore in the present study effect of curcumin on development of drug resistance is being evaluated.

**Methods:**

Male Swiss albino mice (n=40) weighing 25 – 30g were divided into 4 groups of 10 animals each. Group 1 and 2 received phenobarbital (PB, 25 mg/ kg, i.p.) and dimethysulphoxide orally. Animals in groups 3^rd^ and 4^th^ received PB (25 mg/kg), after 90 minutes; curcumin (300 and 1000 mg/kg) was given orally respectively. After 30 minutes, shock challenge was given and the incidence of hind limb tonic extension (HLTE) was recorded. This procedure was followed subsequently for 8 days with increased dose of PB (50 mg/kg) and no shock was given. On 9^th^ and 10^th^ day, the dose of PB (25 mg/kg) was given along with curcumin (300 and 1000 mg/kg) and shock challenge and the incidence of HLTE was noted. Following the above procedure, the mice were euthanized and their brains were removed for estimation of MDA and reduced GSH.

**Results:**

There was no protection against HLTE in mice when treated with curcumin 300 and 1000 mg/kg dose. In both the groups 80% of mice developed resistance. The levels of reduced GSH and MDA remained unchanged with curcumin and were similar to normal control group, and DMSO treated group.

**Conclusions:**

Curcumin in both the doses (300 and 1000 mg/kg) was incapable of protecting mice against development of drug resistant seizures but was able to protect against oxidative stress caused by maximal electroshock challenge.

**272**

**Synergistic effect of *Withania somnifera* dunal and l-dopa in the inhibition of haloperidol-induced catalepsy in mice**

Gupta Girdhari Lal, Rana AC

B. N. College of Pharmacy, Udaipur -313 002 (Rajasthan) India.

The possible synergism between WS and dopamine precursor L-dopa to inhibit haloperidol-induced catalepsy was investigated by using standard bar test in mice. The effect of WS (20-200 mg/ kg, oral), L-dopa (20-200 mg/kg, oral) plus carbidopa (2-20 mg/kg, oral) and combination of subeffective doses of WS (20 or 50 mg/ kg, oral) prior to L-dopa (20, 50 or 100 mg/kg, oral) plus carbidopa was assessed in haloperidol (1 mg/kg, i.p.) induced catalepsy. L-dopa and carbidopa combination was always administered in 10:1 ratio. WS (100 or 200 mg/kg, oral) and L-dopa (50, 100 or 200 mg/kg, oral) plus carbidopa treated groups showed a dose dependent reduction in cataleptic scores. Subeffective doses of WS (20 or 50 mg/kg, oral) prior to L-dopa (20, 50 or 100 mg/kg, oral) also potentiated the anticataleptic effect of L-dopa. These results indicate that subeffective doses of WS enhance the anticataleptic actions of L-dopa and the possibility of using WS as adjunctive therapy to reduce the doses and the adverse effects of dopamine precursor in Parkinson’s disease.

**273**

**Curcumin prevents phenytoin–induced cognitive impairment and oxidative stress in rats**

Reeta KH, Mehla J, Gupta YK

All India Institute of Medical Sciences, New Delhi – 110 029, India.

**Introduction:**

Epilepsy requires long term antiepileptic drug (AED) therapy. Chronic phenytoin therapy has been associated with cognitive impairment. Curcumin has been shown to possess antidepressant, antioxidant and neuroprotective effects among others. The present study investigates the effect of chronic curcumin administration on phenytoin-induced cognitive impairment and oxidative stress in rats.

**Methods:**

Adult male Wistar rats (250-300 g) were administered drugs/vehicle for 21 days. Learning and memory behavior was analyzed using one trial passive avoidance paradigm, elevated plus maze and closed field activity test. On day 21, serum phenytoin concentrations (autoanalyzer) and whole brain malondialdehyde (MDA) and glutathione (GSH) levels were estimated. Data was analyzed by one way analysis of variance (ANOVA).

**Results:**

Phenytoin (75 mg/kg, i.p.) produced significant progressive deficits in learning, memory and cognitive behavior as indicated by the significant impairment in the passive avoidance paradigm and the elevated plus maze. Phenytoin also caused significant oxidative stress as evident from elevations in MDA and reduction of GSH brain levels. When administered with phenytoin, curcumin (100, 200 and 300 mg/kg, orally) significantly prevented phenytoin -induced cognitive impairment and oxidative stress in a dose dependent manner. However, there were no significant differences in the serum levels of phenytoin in any of the treatment groups.

**Conclusions:**

Curcumin is effective in preventing phenytoin-induced cognitive impairment and oxidative stress in rats without altering the serum phenytoin levels. This study suggests the potential of adjuvant curcumin therapy in reducing cognitive impairment by AED therapy.

**274**

**Effects of anti-depressant drugs on behaviour of rats following impact accelerated traumatic brain injury**

Pandey D, Mahesh R, Rajkumar R, Katiyar S

Pharmacy Group, Birla Institute of Technology and Science, Pilani, Rajasthan, India.

**Introduction:**

Traumatic brain injury (TBI) is a disorder of major public health significance. Depression and anxiety are frequent complications of TBI that exert a deleterious effect on the recovery process and psychological outcome in brain injury. In humans, TBI disrupts the neuronal circuits directly including the neurotransmitter systems such as noradrenaline, serotonin, dopamine and acetylcholine which are implicated in depression and anxiety. The effects of anti-depressants on anxiety and depression following TBI in rats were investigated.

**Methods:**

TBI was induced in anesthetized, adult, male wistar rats, using the impact acceleration method. Post ten days of healing, TBI rats were treated with vehicle or anti-depressants (ADs) such as escitalopram, venlafaxine, amitriptyline and bupropion till the second last day of test period. TBI rats were subjected to behavioural anti-depressants and anti-anxiety assays (forced swim test, hyperemotionality, elevated plus maze, social interaction and home cage emergence test), following chronic treatment with ADs.

**Results:**

Behavioural assessments revealed that impact accelerated TBI leads to significant depressive and anxiogenic-like behaviour. Among the various chronic treatments, escitalopram and venlafaxine were most effective in reversing the behavioural anomalies of TBI.

**Conclusions:**

The present findings indicate that chronic treatments with ADs lead to improvement in the depressive/anxiogenic-like behaviour in the TBI rat corroborating the involvement of serotonin and norepinephrine on the behavioural impairment of post- TBI.

**275**

**Rasagiline, a new selective MAO-B inhibitor with neuroprotective effect *in vitro* and in human Parkinson’s disease**

Finberg JPM

Technion, Haifa, Israel.

Rasagiline is the second selective MAO-B inhibitor to be approved for the treatment of Parkinson’s disease (PD), and differs from the other existing inhibitor, selegiline, in not being metabolized to amphetamines. Both drugs can be employed in monotherapy in the early stage of the disease, and also in combined therapy with L-dopa in later stages. In rat brain tissue, the ED50 for inhibition of MAO-B *in vitro* is 4.4 ± 0.92 nM; the potency ratio for inhibition of MAO-B as compared to MAO-A is 1:94 *in vitro*, and 1:65 *in vivo* following oral administration. In the normal rodent brain *in vivo*, dopamine behaves as a MAO-A substrate as shown by microdialysis, but following combined dopaminergic and serotonergic denervation in the rat striatum, rasagiline increases extracellular fluid levels of L-dopa-derived dopamine with reduction in oxidized metabolites. Rasagiline possesses neuroprotective effect *in vitro* as assessed by prolongation of survival of rat fetal mesencephalic and postnatal cerebellar neurons. This action, as studied *in vitro*, is thought to be independent of its MAO inhibitory effect, but its precise molecular target is still unknown. Recently, rasagiline has been found to significantly reduce progression of Parkinson’s disease by delayed-start clinical trial (ADAGIO trial, 2008), which could be the result of MAO- or non-MAO-dependent mechanisms, since oxidative metabolism of dopamine produces toxic byproducts such as hydroxyl free radicals.

**276**

**Effect of cannabinoidergic drugs in the dorsal hippocampus of 3-days apomorphine-treated rats in memory retrieval**

Zarrindast MR^1^^,2^, Nasehi M^1^

^1^Tehran University of medical sciences, Tehran, Iran; ^2^School of Cognitive Sciences (IPM), Tehran, Iran.

In the present study, the effects of injection of cannabinoid receptor agents into dorsal hippocampus (intra-CA1) on memory retrieval have been investigated in 3-days apomorphine-treated rats. Passive avoidance task of memory has been used to examine retrieval 24 h after training. Apomorphine was injected subcutaneously (S.C.), once daily for 3-days followed by 5 days free of the apomorphine before training. Post-training intra-CA1 infusions of the non selective CB1-CB2 receptor agonist, WIN55, 212-2 (0.1, 0.25 and 0.5 *µ*g/rat), dose-dependently shortened the step-through latency, suggesting impaired memory retrieval, whereas posttraining intra-CA1 micro-injections of the selective CB1 receptor antagonist, AM251 (25, 50 and 100 ng/rat) did not affect memory retrieval. Intra-CA1 infusions of AM251 and WIN55, 212-2, tow min apart, did not modify the WIN55, 212-2-induced reduction of stepthrough latency. However, the deleterious effect of WIN55, 212-2 (0.25 *µ*g/rat) was completely abolished in rats previously given apomorphine (0.5 and 1 mg/kg/day, S.C.) for 3 days. This reversal of WIN55, 212-2-induced amnestic-like effect was counteracted by the dopamine D2 receptor antagonist, sulpiride (0.25, 0.5 and 1 mg/ kg/day×3-days, S.C.), administered 30 min before each injection of apomorphine (0.5 mg/kg/day×3-days, S.C.), whereas the D1 receptor antagonist, SCH 23390 (0.01, 0.02, 0.07 and 0.1 mg/ kg/day×3-days, S.C.), was ineffective in this respect. The results suggest that CA1 has an important role in cannabinoid-induced amnesia and subchronic apomorphine treatement may induce dopamine D2 receptor sensitization, which in turn affects CB1 receptor-induced amnesia.

**277**

**Potential of ezetimibe in memory deficits associated with dementia of Alzheimer’s type in mice**

Sharma B, Dalla Y, Kaur S, Kaur H, Singh NJ, Jaggi AS

Department of Pharmaceutical Sciences and Drug Research, Punjabi University, Patiala (Punjab), India.

High cholesterol levels have been positively correlated with higher incidences of memory impairment and dementia. The present study was undertaken to investigate the potential of lipid lowering drug, ezetimibe, in memory deficits associated with dementia of Alzheimer’s (AD) type in mice. Dementia was induced with chronic administration of high fat diet (HFD) or intracebroventricular Streptozotocin (ICV STZ, two doses of 3mg/kg) in separate groups of animals. The memory of the animals was assessed by employing Morris water maze. Brain thio barbituric acid reactive species and reduced glutathione levels were measured to assess total oxidative stress. Brain acetyl cholinesterase (AChE) activity and total serum cholesterol levels were also measured. STZ / HFD produced a significant impairment of memory along with increase in brain AChE activity and oxidative stress. HFD mice also showed an increase in cholesterol levels. Ezetimibe (10 mg/ kg, orally for 15 days) significantly attenuated STZ / HFD induced memory deficits and biochemical changes. It also prevented HFD induced rise in cholesterol level. The memory restorative effect of ezetimibe may be attributed to its cholesterol dependent as well as cholesterol independent effects. The study highlights the potential of ezetimibe in memory dysfunctions associated with dementia of AD.

**278**

**CNS activity of some heterocyclic compounds**

Kadam P, Kadam G, Somani R, Ghosh R, Vohra R, Kadam VJ

Bharati Vidyapeeth’s College of Pharmacy, Sector-8, C.B.D. Belapur, Navi Mumbai, India.

1, 3, 4-oxadiazole derivatives are the most used drugs in the pharmacotherapy of Epilepsy. A large number of structurally different classes of drugs are active in treatment of epilepsy and anxiety. Various 1, 3, 4-oxadiazole derivatives have been found to have very potent anticonvulsant activity. This study was undertaken to provide a behavioral characterization of two novel Mannich bases of 3, 5-disubstituted 1, 3, 4-oxadiazole-2-thione. These compounds were tested and compared to diazepam (0.5 mg/kg) and phenytoin (25 mg/kg) as a standard of activity. When injected in mice both synthetic 1, 3, 4-oxadiazole derivatives showed CNS activity and the exploratory skills of the animals are measured in various models of anticonvulsant, antianxiety and sedative hypnotics. Both compounds indeed, had a clear anticonvulsant activity in models i.e. Maximum Electroshock test, pentylenetetrazole induced convulsions and antianxiety activity in models i.e. Staircase test, Elevated plus maze test and other CNS activity in models i.e. Rotarod test, Tail-immersion test, Hole board test, Ketamine induced sleeping time test. At the tested doses, both compounds showed good CNS activity. These result encourage making deeper investigation on this field.

**279**

**Influence of intracerebral administration of no drugs in dorsal hyppocampos (CA1) on chanabinoid state-dependent memory in the step-down passive avoidance test**

Fazli-Tabaei S^1^, Nasehi M^2^, Piri M^2^, Zarrindast MR^2^

^1^Tehran Medical Unit of Azad University, Tehran, Iran; ^2^Tehran University of Medical Sciences, Tehran, Iran.

The present study, effects of nitric oxide agents on WIN55, 212-2 induced state-dependent memory of passive avoidance task were examined in mice. One-trial step-down paradigm was used for the assessment of memory retention in adult male NMRI mice. Posttraining intra-CA1 administration of CB1 receptor agonist, WIN55, 212-2 (0.25, 0.5 and 1 *µ*g/mouse), dose-dependently decreased memory retrieval. The memory impairment by WIN55, 212-2 (1*µ*g/ mouse) was completely reversed by pre-test administration of the same dose of the drug (1*µ*g/mouse, intra-CA1), suggesting WIN55, 212-2 state-dependent memory. Administration of L-arginine (0.3, 1 and 3 *µ*g/mouse) and L-NAME (0.3, 1 and 3 *µ*g/mouse) intra-CA1, 5 min before test by itself could not alter memory retrieval. On the other hands, in the animals in which retrieval was impaired due to WIN55, 212-2 (1*µ*g/mouse) post-training administration, pre-test administration of L-arginine (1 and 3 *µ*g/mouse) and L-NAME (0.3, 1 and 3 *µ*g/mouse) intra-CA1 24 hr after train in day’s test restored and have no effect on retrieval, respectively. On the other hand injection L-NAME (3 *µ*g/mouse, intra-CA1) 2 min before administration of WIN55, 212-2 (1*µ*g/mouse) in day’s test in animal that received post training WIN55, 212-2 (1*µ*g/mouse) block Stat-dependency. In the other group administration of receiving post-training WIN55, 212-2 (1*µ*g/mouse), combined pre-test non effective doses of WIN55, 212-2 (0.25 *µ*g/mouse) and L-arginine (0.3 *µ*g/mouse) administration increased the restoration of memory by the WIN55, 212-2. These findings implicate the involvement of a dorsal hippocampal nitric oxide mechanism in the cannabinoid state-dependent memory and also it can be concluded that for state-dependency of cannabinoid increase level of NO in CA1 is necessary.

**280**

**Effect of evolvolus alsinoides root extract on acute reserpine induced orofacial dyskinesia**

Muralidharan P, G Manoj

C.L.Baid Metha College of Pharmacy, Chennai-97, India.

**Introduction:**

Tardive dyskinesia is a neurological syndrome caused by the long-term use of neuroleptic drug. Repetitive, involuntary, purposeless movements characterize it. Features of the disorder may include grimacing, tongue protrusion, lip smacking, puckering, pursing and rapid eye blinking. Rapid movements of the arms, legs and trunk may also occur

**Methods:**

*In vivo* pharmacological evaluation of methanolic extract of *Evolvuls alinoides* roots (MEEA) was evaluated for its behavioral assessment in a plexi glass cage for its Vacous chewing movements and tongue protrusion. The two major symptoms of orofacial dyskinesia (VCMs) and tongue protrusion (TP) were produced in experimental rats by acute reserpine (1.0 mg/kg, s.c.) administrations. Cognitive behavior was assessed by the using the elevated plus-maze learning task, which measures the spatial long-term memory; the locomotor activity was monitored using an actophotometer.

**Results:**

In the present study acute reserpine treated animals showed increased frequencies of VCMs and TPs compared with vehicle treated animals. Chronic treatment with MEEA significantly reversed the reserpine induced VCMs and TPs in a dose dependent manner and also the locomotor activity. Chronic MEEA administration significantly decreased the transfer latency in acute reserpine treated rats.

**Conclusions:**

Methanolic extract of *Evolvuls alinoides* root extract showed significant effect in the reversal of drug induced Tardive Dyskinesia and memory dysfunction.

**281**

**Neuroprotective effect of carvedilol, an adrenergic antagonist against cognitive impairment and oxidative stress in an animal model of sporadic demetia of Alzheimer’s type**

Prakash Atish, Kumar Anil, Dogra Samrita

Pharmacology Division, University Institute of Pharmaceutical Sciences, Panjab University, Chandigarh – 160014, India.

Alzheimer’s disease is a progressive neurodegenerative disorder associated with cognitive impairment and weak intellectual capacity. Growing evidences indicate that oxidants and antioxidant defenses interact in a vicious cycle, which plays a central role in the pathogenesis of Alzheimer’s disease. The present study was carried out to elucidate the neuroprotective effect of carvedilol against the colchicine-induced cognitive impairment and oxidative damage in rats. Colchicine (15*µ*g/5*µ*l), a microtubule disrupting agent when administered intracerebroventricularly in rats resulted in poor memory retention in both Morris water maze, elevated plus maze task paradigms and caused marked oxidative stress as indicated by significant increase in malondialdehyde, nitrite levels, depletion of SOD, catalase, glutathione-s-transferase activity and reduced glutathione levels. It also caused a significant increase in the acetylcholinesterase activity. Chronic administration of carvedilol (2.5 and 5.0 mg/kg; p.o.) for a period of 25 days, starting 4 days prior to colchicine administration resulted in an improvement in memory retention, attenuation of oxidative damage and marked reduction of acetylcholinesterase activity. Present study demonstrates a neuroprotective effect of carvedilol against colchicine-induced cognitive impairment and associated oxidative damage.

**282**

**Hypertension is a major modifiable risk factor for acute ischemic stroke – a experimental study in spontaneous hypertensive rats**

Thakkar P^1^, Patel R^1^, Patel M^1^, Patel N^2^

^1^K. B. Raval College of Pharmacy, Shertha, Gujarat, ^2^S. K. Patel College of Pharmaceutical education and Research, Kherva, Mehsana, India.

Strokes are a heterogeneous group of disorders caused by interruption of cerebral blood flow to the parts of brain and responsible for severe long term disability and mortality. Stroke has many modifiable risk factors like hypertension, diabetes, cigarette smoking. Epidemiological data suggests that most number of stroke affected patients represent such hypertensive condition and stroke when occurs along with this disease would have very severe outcome. Hypertension is related to the higher incidence of mortality and lead to stroke but very few preclinical neuroprotective studies have conducted in animal models with Hypertensive associated with stroke condition. This fact is a probable reason for the failure of many neuroprotective clinical trials. In experimental study four groups (8 male animals in each group) were designed [2 for Wistar rats (Surgery and Sham) and 2 for SHR rats (Surgery and Sham)] and intraluminal filament technique was used to induce stroke. All animals are male animals and their body weight was 300-350 gm. Blood pressure was measured by NIBP instrument for all groups and animals were taken for study if they had blood pressure >160 mmHg for SHR animals. After 7 days, live animals were sacrificed and brain slices were taken by matrix machine. % Infarct, % Edema, Neurological deficit, % Weight loss were calculated from statistical and computer software. All parameters showed severe outcome and mortality in hypertensive group. So it is worth testing putative neuroprotective agents in such stroke models with disease conditions before the molecules enter the costly clinical trials.

**283**

**Pyrimidine analogue 1(4- nitrophenyl)4, 4, 6 trimethyl, (1H, 4H) pyrimidine – 2 thiol: A potential new antiepileptic**

Bansal V^1^, Verma P^2^, Walia R^2^

^1^Post Graduate Institute of Medical Education and Research, Chandigarh, India; ^2^Government Medical College, Patiala, India.

**Introduction:**

Epilepsy is a disease with significant morbidity with no effective cure despite availability of a large number of antiepileptic drugs. Pyrimidine derivatives have a wide range of therapeutic uses with most of the pyrimidine compounds having some central nervous system activity. 1(4- nitrophenyl) 4, 4, 6 trimethyl, (1H, 4H) pyrimidine – 2 thiol, is a new pyrimidine analogue with structural resemblance to phenobarbitone.

**Methods:**

It was studied at different dose levels (10, 20, 40 and 80 mg/ kg body weight) for its effects on MES (maximal electroshock seizure) and PTZ (pentylene tetrazole) induced convulsions in mice and compared with equivalent doses of phenobarbitone.

**Results:**

With the test compound, for PTZ induced convulsions, 20 mg, 40 mg and 80 mg per kg body weight dose produced mean percentage protection of 48.34% (*P*< 0.05), 60% (*P*<0.05) and 90% (*P*<0.001). Mean percentage protection for MES seizure was 30%, 41.67%, 42.5% and 81.67% with doses of 10, 20, 40 and 80 mg/kg (*P*<0.05) respectively. The mean percentage protection offered by phenobarbitone for PTZ induced convulsions was 53.4% and 61.67% at 40 and 80 mg/kg body weight (*P*<0.05 for these doses). MES at doses 10, 20, 40 and 80 mg/kg body weight of mice produced mean percentage protection of 48.34%, 60%, 80% and 98.34% respectively, which was significant (*P*<0.05) at all dose levels. Discussion: the test compound produced significant protection in both MES and PTZ induced convulsions.

**Conclusions:**

This new pyrimidine analogue may thus be potentially tested for its further activities in epilepsy.

**284**

**Cerebroprotective effect of methanolic root extract of asparagus racemosus in cerebral ischemia induced by bilateral carotid artery occlusion in rats**

Muralidharan P, Nandagopal M

C.L.Baid Metha College of Pharmacy, Chennai-97, India.

**Introduction:**

Stroke is a life-threatening disease characterized by rapidly developing clinical sign of focal or global disturbance of cerebral function due to cerebral ischemia The incidence of brain infarction, following a reduction of blood flow, is gradually increasing due to relatively recent life style changes, such as consumption of fatty foods, smoking and excess stress.

**Methods:**

In the present study the animals were pretreated with methanolic root extract of asparagus racemosus for a period of 1week (200 and 400 mg/kg) i.p, The animals were anaesthetized with thiopental sodium (45mg/ kg) and stroke was induced by occlusion of bilateral carotid artery (BCAO) for 10 min with aneurism clips placed on both arteries and 10 min later clips were removed to allow reperfusion and animals were then returned to their cages. After 24 hours of reperfusion the neurological functions were evaluated. The treatment was continued for another week after surgery with root extract and the animals were sacrificed and the brain was removed and homogenized. The homogenized content was used for the estimation of brain super oxide dismutase (SOD), malonyldialdehyde(MDA), glutathione(GSH) and glutamate neurotransmitter levels.The infarct volume was been assed by 2, 3, 5 triphenyltetrazolium chloride staining.

**Results:**

Pretreatment with root extract at 200 and 400mg/kg significantly improved the neurological symptoms, significantly increased SOD levels, levels of brain MDA were significantly attenuated, levels of GSH also significantly increased, neurotransmitter glutamate level was significantly reduced and infarct volume was significantly reduced in dose dependent manner in pretreated BCAO group compared to vechicle treated BCAO group.

**Conclusions:**

Asparagus racemosus root extract showed significant cerebroprotective effect in cerebral ischemia induced by bilateral carotid artery occlusion in rats.

**285**

**Memory enhancing effect of *Asparagus racemosus* and *Phyllanthus emblica***

Ashwlayan VD^1^, Singh M^2^

^1^University of Shobhit, School of Pharmaceutical Sciences, Meerut, India; ^2^ISF Institute of Pharmaceutical Sciences and Drug research, Moga, India.

The root extract of *Asparagus racemosus* Wild (Shatavari) is reported to increase the formation and release of estrogens in brain (Handa, 1996). It is also suggested to rejuvenative, loss of libido (Rege *et al*, 1999) and impotency (Roy *et al*, 1971). *Phyllanthus* emsblica Linn (Syn: *Emblica officinalis* Gaertn., Emblic myrobalan) fruit is not only useful as anti-oxidant (Chaudhuri, 2002) and anti-inflammatory (Roy *et al*, 1991), but had adaptogenic activity (Ying et al, 2004). It is used clinically for degeneration caused by Alzheimer’s disease. Thus it is speculated that these two plants may be useful to relieve amnesia. The methanolic extract of *Asparagus racemosus* Wild root and *Phyllanthus emblica* Linn.fruit were prepared and concentrated to obtain dark viscous masses. It was further fractionated using nonpolar to polar solvents. Scopolamine (0.4 mg Kg^-1^, i. p.) and sodium nitrite (1 mg Kg^-1^, i. p.) induced amnesia in mice, were employed to evaluate the effect of each fraction obtained from each plant. The methanolic fraction of both the plants extracts significantly reversed NaNO_2_ and scopolamine induced retrograde, induced anterograde amnesia. From HPLC and HPTLC chromatographic profile of the methanolic fraction of Aspragus racemosus roots and *Phyllanthus emblica* fruits has been investigated for chemical constituents. It may be speculated that memory enhancing effect of *Asparagus racemosus* and *Phyllanthus emblica* may be due to their antioxidant effect, polyphenolic compounds and ascorbic acid leading to rejuvenation of nervous system.

**286**

**Pharmacological evaluation of anti-stress activity of roots of *Boerhaavia diffusa***

Desai SM, Sanaye M, Desai SK, D’souza LL

Prin. K. M. Kundnani College of Pharmacy, Mumbai, India.

**Introduction:**

The incidence of toxicity and dependence often seen with synthetic drugs has limited their therapeutic usefulness in stress and are substituted by cheaper, safer herbal medicines. *Boerhaavia diffusa* (Punarnava) family Nyctaginaceae is an important indigenous medicine for the treatment of jaundice and stress.

**Materials and Methods:**

The activity of hydroalcoholic extract of *B. diffusa* was assessed at two dose levels-150mg/kg and 300 mg/kg to evaluate the antistress activity of HEBD- (i) Cold forced swim test and (ii) Tail suspension test. Parameters evaluated on Day 7 for both models: A) SGPT, SGOT, ALP, cholesterol, glucose, triglycerides. b) Changes in the weight of spleen. Ashwagandha (reference standard).

**Results:**

HEBD showed a significant reduction in stress induced increased levels of all the above parameters. In FST, 300 mg/kg showed extremely significant results for ALP (119±24.72 IU/L) and glucose (63.62 3.94 IU/ L) as compared to control. In TST, with 300 mg/kg significant reduction in ALP (49 0.675), SGOT (136.12 0.692) and SGPT (31.63 3.18) in IU / L units respectively was seen as compared to control. The duration of immobility of the mice and their spleen weight were reduced in treated groups.

**Conclusions:**

The present study is indicative of *Boerhaavia diffusa* possessing good anti-stress activity and warrants further experimentation to understand its mechanism of action.

**287**

**Screening of selected Indian plants for their antiepileptic potential in PTZ and MES induced seizures in rats**

Pahuja M^1^, Mehla J^1^, Reeta KH^1^, Tripathi VK^2^, Gupta YK^1^

^1^All India Institute of Medical Sciences, New Delhi, India; ^2^Sanat Products Ltd., Delhi, India.

**Introduction:**

In the present study hydroalcoholic extracts of nine Indian plants that are used traditionally for neurological disorders such as Alzheimer’s disease, convulsions and depression were tested for their antiepileptic potential in pentylenetetrazole (PTZ) - and maximal electroshock (MES)-induced seizures in rats.

**Materials and Methods:**

Hydroalcoholic extracts of *Anacyclus pyrethrum, Argyreia nervosa, Benincasa hispida, Caesalpinia sappan, Cinnamomum camphora, Mucuna pruriens, Nepeta hindostana, Orchis mascula,* and *Zizyphus jujube* were evaluated in a dose of 1 g/kg. Each group consisted of 6 animals. PTZ (60 mg/kg i.p) was administered to each rat 30 min. after the administration of plant extracts. The latency of myoclonic jerks and incidence of generalized tonic clonic seizures with loss of righting reflex were noted. MES (current intensity of 70 mA at fixed frequency –299 Hz and duration- 0.2s.) seizures were induced in rats 30 min. after the administration of plant extracts using ECT unit. The animals were observed for the latency and duration of tonic hind limb extension (THLE). Valpraote and phenytoin served as positive controls in PTZ and MES models, respectively.

**Results:**

Hydroalcoholic extracts of *Anacyclus pyrethrum, Benincasa hispida, Orchis mascula* and *Zizyphus jujube* showed 0% incidence of GTC and a seizure scores of 0.16, 0.62, 0.74 and 0.41 respectively, in PTZ induced seizures. Hydroalcoholic extract of Nepeta hindostana showed 100% protection against THLE in MES induced seizures.

**Conclusions:**

The results from the preliminary studies show that the hydroalcoholic extracts of *Benincasa hispida, Orchis mascula, Zizyphus jujube* and *Nepeta hindostana* are potential candidates in the treatment of epilepsy.

**288**

**Neuro protective effect of *Ficus hispida* linn on β-amyloid induced cognitive dysfunction**

Sivaraman D, Muralidharan P

C.L. Baid Metha College of Pharmacy, Chennai-97, India.

**Introduction:**

Alzheimer’s disease (AD) is the most common of the senile dementias. It is estimated that 14 million affected worldwide by 2025. AD is a progressive, neuro degenerative disease characterized by memory loss, language deterioration, poor judgment, impaired visuospatial skills, etc. Dysfunction of cholinergic neurotransmission in the brain contributes to the salient cognitive decline in AD. Amyloid β protein (Aβ) may be neurotoxic during the progression of Alzheimer’s disease by eliciting oxidative stress.

**Methods:**

The present study was designed to determine the effect of *Ficus hispida* Linn methanol extract (FME) leafs (collected from Eastern Ghats of South India) on Aβ25-35-induced cognitive deficits and oxidative stress in mice. Animals were treated with FME for periods of 4 weeks dose-dependently (200 and 400 mg/kg) then received a single intra cerebro ventricular (i.c.v.) injection of Aβ25- 35 (10*µ*g/mouse). Behavioral changes in the mice were evaluated using passive avoidance, Y-maze, Plus maze and water-maze tests. Anti-oxidant enzymes and neuro-transmitter levels were also been estimated.

**Results:**

FME at the dose of 400mg/kg significantly ameliorated the cognitive and memory deficits caused by i.c.v. injection of Aβ25-35. FME attenuated the Aβ-induced increase in brain levels of thiobarbituric acid reactive substances. There was an increase in glutathione peroxides, glutathione reductase and super oxide dismutase activity in FME-treated groups. The acetyl cholinesterase activity in the brain was lower in FME supplemented groups than in the only Aβ-injected group. FME treated group showed a significant alteration in behavior when compare to negative control in Y maze, Plus- maze and also in water maze tests. Analysis was done using Dunnett’s t-test, Two- way ANOVA.

**Conclusion:**

These findings suggest FME exerts a protective effect against cognitive deficits induced by Aβ25-35 accumulation in Alzheimer’s disease, because of its potential antioxidant property.

**289**

**Investigation of *Sapindus trifoliatus* on animal models for OCD: Involvement of dopaminergic and serotonergic system**

Srikanth J, Muralidharan P

C.L. Baid Metha College of Pharmacy, Chennai, India.

**Introduction:**

Obsessive-compulsive disorder (OCD) is a devastating culmination of anxiety disorders. OCD usually involves having both obsessions and compulsions. The underlying path physiology of OCD was due to the hypersensitivity in the postsynaptic 5-hydroxytryptamine (5-HT) receptors and the abnormalities in the serotonin system.

**Methods:**

In the Present study *Sapindus tifoliatus* (ST) at 20 and 100 mg/kg, i.p doses was evaluated for its effect on Marble burying Behavior, Quinpirole induced Compulsive Checking, Apomorphine-induced climbing behavior and 5-hydroxytryptophan (*l*-5-HTP)-induced serotonin syndrome. Quinpirole hydrochloride was dissolved in physiological saline and injected under the nape of the neck subcutaneously at a dose of 0.5 mg/ kg (0.5 mg/kg twice weekly for 5 weeks) to induce compulsive checking behavior.

**Results:**

5 weeks) to induce compulsive checking behavior. Results: ST (100 mg/kg, i.p.) significantly inhibited the number of Marbles buried in dose dependant manner significantly as compared to standard drug fluoxetine without affecting the Locomotion. It also inhibited the frequency of stops in each locale (place or object); mean time interval between two successive visits to a given locale; mean duration of stopping in a given locale; the number of visits to other locales in between returns to a given locale in Quinpirole sensitized Rats. ST (100 mg/kg, i.p.) significantly reduced all the five behavioral syndromes induced by *l*-5-HTP; however ST 20 mg/kg inhibited only *l*-5-HTP-induced head twitches. ST (20 and 100 mg/kg, i.p.) significantly inhibited Apomorphine-induced climbing Behavior in mice.

**Conclusion:**

In the present investigation, ST exhibited pharmacological actions mediated through dopamine and serotonin receptors in the central nervous system.

**290**

**Perindopril improves memory decline in rat: Involvement of angiotensin converting enzyme in memory deficit induced by ICV streptozotocin**

Tota S, Kamat KP, Nath C, Hanif K

Central Drug Research Institute (CDRI), P.O. Box 173, Lucknow (U.P.) 226001, India

**Introduction:**

The Renin-angiotensin system, besides blood pressure regulation, affects learning and memory as evidenced by improvement of cognition in hypertensive patients being treated with angiotensin converting enzyme inhibitor (ACEI). Methods: The present study examined the influence of an ACEI, perindopril, on memory impairment induced by ICV streptozotocin (ICV STZ 0.5 mg/kg) in rat. Perindopril (0.05 mg/kg and 0.1 mg/kg, orally) was given for fourteen days following ICV STZ administration. After fourteen days, the rats were subjected to Morris water maze test. Results: Treatment with both doses of candesartan for fourteen days significantly improved spatial memory in rats in water maze test. Conclusion: These results suggest that angiotensin converting enzyme play a facilitatory role in STZ induced memory deficit and corroborate number of human studies that angiotensin converting enzyme inhibitor can be used therapeutically against cognitive decline in hypertensive patients.

**291**

**Nitric oxide and obsessive-compulsive behavior in mice: Modulation by paroxetine**

Utturwar KS, Shukla NR, Borkar SS, Bhutada PS, Umathe SN

Department of Pharmaceutical Sciences, Rashtrasant Tukadoji Maharaj Nagpur University, Nagpur - 440 033, Maharashtra, India.

**Objective:**

In view of the fact that plasma nitrite levels are higher in obsessive-compulsive disorder, nitric oxide modulates the neurotransmitters implicated in this disorder and paroxetine- a SSRI, influences the NO turnover, it was proposed to investigate in mice the probable role nitric oxide in obsessive-compulsive behavior and in anti-compulsive effect of paroxetine.

**Materials and Methods:**

The obsessive compulsive behaviour in mice was assessed by marble burring behaviour in different groups that were treated with various NO modulators prior to paroxetine or vehicle. The treatments included NO ehhancers such as sodium nitroprusside (1-3 mg/kg i.p.) and sildenafil (1-3 mg/kg i.p.), while nNOS inhibitor was 7-nitroindazole (10-40 mg/kg i.p.) and anti-compulsive agent paroxetine (2.5-10 mg/kg, i.p). The concentration of nitric oxide in brain homogenate was estimated after each treatment.

**Results:**

Sodium nitroprusside (3 mg/kg) or sildenafil (3 mg/kg) significantly increased marble-burying behavior and brain nitric oxide levels, whereas 7-nitroindazole (20-40 mg/kg) paroxetine (5-10 mg/kg, i.p.) dose dependently attenuated marble-burying behavior and nitrites levels in brain. Further, co-administration of sub-effective doses of 7-nitroindazole (10 mg/kg) and paroxetine (2.5 mg/kg) significantly attenuated marble-burying behavior. Moreover, pre-treatment with sodium nitroprusside (2.0 mg/kg, i.p.) or sildenafil (2.0 mg/kg, i.p.) significantly attenuated the inhibitory influence of 7-nitroindazole (40 mg/kg) or paroxetine (10 mg/kg) on marble-burying behavior and brain nitrite level. All the behavioural effects were devoid of any influence on locomotor activity.

**Conclusions:**

Present study indicates the involvement of nitric oxide in obsessive-compulsive behaviour, and paroxetine produces its anti-compulsive effect via its inhibitory influence on nitric oxide turnover in brain.

**292**

**Comparative study of efficacy, safety and cognitive profile of amisulpride and olanzapine in the treatment of acute psychotic exacerbations of schizophrenia**

Pawar GR^1^, Agarwal RP^1^, Phadnis P^1^, Solanki P^1^, Paliwal A^2^

^1^MGM MC Indore, M.P., India; ^2^MY Hospital, Indore, M.P., India.

**Introduction:**

Schizophrenia is a common psychiatric disorder characterized by positive symptoms [hallucinations, delusions], negative symptoms [anhedonia, apathy] along with cognitive dysfunctions in areas of memory and executive functions. Conventional antipsychotics have a modest benefit in improving cognitive dysfunctions. Also, current treatment of schizophrenia with these typical antipsychotics is limited by the side effects of the drugs. Side effects like parkinsonian syndrome, tardive dyskinesia occur in significant number of patients only to deteriorate the quality of life. Amisulpride is an atypical antipsychotic with selective affinity for D2 and D3 receptors with efficacy in positive and negative schizophrenic symptoms with low incidence of extra pyramidal symptoms. This drug also shows improvement in neuropsychological performance in patients of schizophrenia.

**Methods:**

A prospective, randomized, double blind, single center, 8 weeks clinical trial. Subjects and treatments- Seventy-four patients were treated for two months with either amisulpride [400-800 mg/d] or olanzapine [10-20 mg/d].

**Results:**

Brief psychiatric rating scale [BPRS] was used as primary measure of efficacy. Other measures of efficacy and safety were also evaluated. Both amisulpride and olanzapine groups showed equivalent improvement in psychotic symptoms on BPRS scale. Less than five-percent patients suffered adverse effects only to withdraw from the study. Olanzapine group showed statistically significant [*P*< 0.05] weight gain as compared with amisulpride group. Amisulpride group showed significant improvement [*P*<0.05] in various cognitive parameters as compared to olanzapine group.

**Conclusions:**

Amisulpride and olanzapine showed equivalent efficacy in terms of improvement in psychotic symptoms. Amisulpride offers significant advantage in preserving body weight and improvement of cognitive dysfunctions.

**293**

**Effect of piracetam and neuroactive steroids on ischaemia and reperfusion-induced cerebral injury**

Mehta G^1^, Singh M^2^

^1^Raj Kumar Goel Institute of Technology, Ghaziabad, India; ^2^ISF College of Pharmacy, Moga, India.

The present study is designed to investigate the effect of piracetam, estradiol valerate, diethylstilbestrol on ischaemia and reperfusioninduced cerebral injury. Global cerebral ischaemia was induced in mice by occluding both carotid arteries for 10 min followed by reperfusion for 24 hr. Mice were bilaterally ovariectomized to deplete endogenous steroids one week before subjecting them to global cerebral ischaemia. Cerebral infarct size was estimated using triphenyl- tetrazolium chloride staining. Mitochondrial TBARS assay was employed as an index of oxidative stress. Elevated plus maze was employed to estimate short term memory. Degree of motor incoordination was evaluated using inclined beam-walking test and lateral push test. Global cerebral ischaemia followed by reperfusion produced a significant impairment in short term memory and motor coordination and produced a notable increase in mitochondrial TBARS. Administration of piracetam before and after cerebral ischaemia markedly reduced cerebral infarct size and attenuated impairment in short-term memory and motor coordination. Ovariectomy has markedly enhanced cerebral ischaemia and reperfusion induced neuronal injury. Administration of estradiol valerate and diethylstilbestrol before cerebral ischaemia markedly reduced ovariectomy-induced cerebral infarct size and attenuated impairment in short-term memory and motor coordination. The protective effect of piracetam, estradiol valerate and diethylstilbestrol was accompanied by marked decrease in TBARS. The noted neuroprotectve effect of piracetam, estradiol valerate and diethylstilbestrol may be due to scavenging of reactive oxygen species generated as a result of ischaemia and reperfusion. Moreover, the neuroprotectve effect of estradiol valerate and diethylstilbestrol may be due to restoration of ovariectomy-induced decrease in neurosteroids.

**294**

**Study of the antinociceptive activity of antidepressants (SSRIs and atypical antidepressants) and their interaction with morphine and naloxone in mice**

Sikka P, Kaushik S, Kumar G

M.L.B.Medical College, Jhansi, India.

**Introduction:**

Antidepressant particularly tricyclics and SSRIs have been shown to have analgesic activity both in experimental and clinical pain states. However, the exact mechanism of analgesic activity remains to be fully understood. Therefore, the present study was planned to confirm the antinociceptive activity of SSRIs {fluoxetine (Fl) and escitalopram (Ec)} and atypical Antidepressants {venlafaxine (Ve) and mirtazepine (Mt)}.

**Materials and Methods:**

Study was conducted on albino mice (25-35 grams) and rats (80-100 grams) of either sex using Tail Flick (TFl) and Abdominal Writhing Test (AWT). Subanalgesic doses of Morphine(0.5and1mg/kg), Fluoxetine(2, 5and10mg/kg), Venlafaxine(30, 40and50mg/kg), Mirtazepine(3, 5and7mg/kg) and Escitalopram(2.5, 5 and10mg/kg) were obtained. In tail Flick test, tail flick latencies were obtained 15, 30, 60 and 120 min. after drug administration. Naloxone was administered 10 minutes prior to test drug to test antagonism. In AWT, animals were administered with test drug followed by i.p. injection of 4% Saline (1ml/kg) as irritant 10 min. later. Abdominal Writhing was noted between 30 sec. and 3 min. after saline injection. All drugs were administered by sc route.

**Results:**

in both the tests, fluoxetine (5 and 10mg/ kg), mirtazepine (5 and 7mg/kg), venlafaxine (40 and 50mg/kg) were found to have antinociceptive activity but not at lower doses. Escitalopram failed to show any antinociceptive activity at any of the doses used. The analgesic/ antinociceptive effect of all the drugs was antagonized by naloxone (1mg/kg). Further, suboptimal doses of Fl, Mt and Ve showed analgesic activity with suboptimal dose of Morphine (0.5mg/kg).

**Conclusions:**

Fl, Mt and Ve have antinociceptive activity whereas Ec doesn.t have and their site of action seems to be same as that of Opioid analgesics (‘mue’ receptors).Results apparently show that these drugs may be useful in the management of pain as monotherapy or in combination with other Opioids.

**295**

**Amelioration of intracerebroventricular streptozotocin induced cognitive dysfunction and oxidative stress by vinpocetine- a PDE1 inhibitor**

Sharma V, Gangwani S, Deshmukh R, Bedi KL

I.S. F. College of Pharmacy, Moga, Punjab-142001, India.

**Objective:**

To evaluate the role of Phosphodiestrase 1 inhibition by Vinpocetine in cognitive dysfunction and oxidative stress induced by intracerebroventricular (ICV) STZ in rats.

**Materials and Methods:**

Present study was conducted on male wistar rats (250-280g). Streptozotocin was given intracerebroventrically (3mg/ kg), bilaterally on days 1 and 3 to induce experimental dementia resembling Alzheimer’s type. The rats were treated chronically with Vinpocetine (5, 10 and 20 mg/kg) for 21days starting 1st day of streptozotocin administration. Learning and memory was assessed with Morris water maze and Modified elevated plus maze task paradigms and locomotor activity was examined with Actophotometer. Brain acetylcholinestrase activity was measured by Ellman method. Malonialdehyde, Ntrite and Glutathione levels were measured by Wills *et al*., Ellman *et al*., and Green *et al*., respectively. Lactate dehydrogenase (LDH) was estimated with commercially available kit and total protein content was measured by Biuret method

**Results:**

ICV STZ treated rats exhibited poor retention of memory in Morris water maze and Modified elevated plus maze task paradigms. Chronic treatment with vinpocetine dose dependently improved learning and memory impairment in MWM and EPM tasks. Vinpocetine showed potent antioxidant effect by significantly reducing elevated levels of Malondialdehyde and nitrite and restoring reduced levels of glutathione. It also reduced raised levels of AChE and LDH induced by STZ indicating its effect on cholinergic function and neuronlal viability.

**Conclusion:**

Present study strongly support candidature of vinpocetine as a neuroprotective agent, involvement of overexpression of phosphodiestrases and role of secondary messangers(cAMP and cGMP) in dementia of Alzheimer’s type

**296**

**Anticonvulsant investigation of some substituted aryl semicarbazones by maximal electroshock seizure test model**

Acharya PC, Raja AS, Putta A

Banaras Hindu University, Varanasi, India.

**Introduction:**

Semicarbazones (>C=N-NHCONH-R) have been identified as novel templates for the development of new chemical entities for the treatment of central nervous system disorders and infectious diseases. In our laboratory, large number of semicarbazones have been synthesized and evaluated for their anticonvulsant activity. In continuation of our search on potential anticonvulsant leads, six substituted aryl semicarbazones (compound no. I-VI) were synthesized and evaluated for their activity.

**Methods:**

Maximal electroshock seizure (MES) test model in albino rats (180-250gms) was used to screen the synthesized compounds. Maximal seizure was induced by application of an electrical current across the brain through corneal electrodes. The stimulus parameters were 50 Ma, AC in a pulse of 60 Hz for 200 Ms (0.2 sec). Animals giving positive hind limb extensor response were taken for testing drug substances. After 24 hours, animals were administered with test drug solution intraperitoneally. Maximal seizure was induced after 0.5 hr and 4 hr of drug administration and abolition of hind limb tonic extensor spasm was recorded as a measure of anticonvulsant activity. Phenobarbitone was taken as the standard drug.

**Results:**

Three compounds (I, II and III) showed excellent activity with 100% seizure protection while other three (IV, V and VI) had moderate activity with 50% seizure protection efficiency.

**Conclusion:**

The test results indicated that the synthesized compounds have potent anticonvulsant activity. Further studies are under progress to establish their potency and future prospective for the development of these molecules as novel therapeutic agents for the treatment of epilepsy.

**297**

**Possible involvement of L-type calcium channels and ryanodine receptors in digoxin preconditioning induced cerebroprotection in mice**

Reetu, Kaur S, Singh N, Jaggi AS

Department of Pharmaceutical Sciences and Drug Research, Punjabi University, Patiala (Punjab), India.

The present study was designed to investigate the possible neuroprotective effect of digoxin induced pharmacological preconditioning (PP) and its probable mechanism. Bilateral carotid artery occlusion (BCAO) of 17 min followed by reperfusion for 24 h was employed to produce ischemia and reperfusion (I/R) induced cerebral injury in male swiss albino mice. Cerebral infarct size was measured using triphenyltetrazolium chloride staining. Memory was assessed using elevated plus maze test. Degree of motor incoordination was evaluated using inclined beam walking test, rota rod test and lateral push test. Digoxin (0.08 mg/kg, i.p.) was administered 24 h before surgery in a separate group of animals to induce PP. BCAO followed by reperfusion, produced significant rise in cerebral infarct size along with impairment of memory and motor coordination. Digoxin treatment produced a significant decrease in cerebral infarct size and reversal of I/R induced impairment of memory and motor incoordination. Digoxin induced neuroprotective effect was abolished significantly by verapamil (15 mg/kg, i.p.), a L-type calcium channel blocker, and ruthenium red (3 mg/kg, s.c.), an intracellular ryanodine receptor blocker. These findings indicate that digoxin preconditioning exerts a marked neuroprotective effect on the ischemic brain, which is possibly linked to digitalis induced increase in intracellular calcium levels eventually leading to the activation of calcium sensitive signal transduction cascades.

**298**

**Light replenishing effect of vitamin D in dark induced depression**

Bhatia M, Mittal M, Goel RK

Punjabi University, Patiala, India.

Seasonal Affective Disorder is most commonly linked to decrease in photoperiod either naturally (as during Winter season) or artificially (as in case of people working for most part of the day in closed, non natural light environment). Length of photoperiod is also linked to biosynthesis of vitamin D in the body. Therefore the present study was carried out with the hypothesis that Vitamin D may ameliorate dark induced depression. Albino Swiss mice adapted to 12h: 12 h light: dark cycle (Light Dark group) were kept in 12h: 12 h Dark: dark cycle (Dark Dark group) for 5 days to induce depression. Vitamin D (250, 500, 1000 IU/Kg) and Fluoxetine (20mg/Kg) were administered intra-peritoneally once daily during dark treatment followed by tail suspension test (TST) and forced swimming test (FST) to determine the effect of drug treatment. Brain serotonin (5HT), nor-epinephrine (NE) and serum nitrite/nitrate levels were also determined. Dark treatment induced significant immobility in FST and TST showing CNS depression, which was further supported by reduced brain contents of the 5HT and NE and raised serum nitrite/nitrate levels. Vitamin D supplementation significantly decreased the immobility in FST and TST. Further the brain contents of the 5HT and NE was raised and serum nitrite/nitrate levels were decreased as compared to dark treated group. Thus it may be concluded that Vitamin D shows light replenishing effect for alleviating dark induced depression, which may be mediated via monoaminergic mechanisms.

**299**

**Effect of alprazolam on anxiety and cardiomyopathy induced by doxorubicin in mice**

Anwar MJ, Pillai KK, Khanam R, Akhtar M, Abdullah SM, Vohora D

Jamia Hamdard (Hamdard University), New Delhi, India.

Anxiety and depression following myocardial infarction (MI) and/ or heart failure (HF) can impede recovery and constitutes a major risk factor for further cardiac events. There is considerably less literature on anxiety (than on depression) post MI or HF even though anxiety and depression are highly co-morbid disorders and the prevalence of anxiety may be as high as 60% in patients with HF. The present study was aimed to evaluate anxiety following doxorubicin-induced cardiomyopathy, a rodent model of HF, in mice. Further, the study investigated the effects of alprazolam on anxiety and cardiomyopathy in this model. Swiss strain albino mice were used. Doxorubicin (DOX) (10 mg/ kg, iv) was used for inducing cardiomyopathy in mice. Alprazolam (0.25, 0.5 and 1 mg/kg po) was given pre (7 days) and post (7 days) doxorubicin. Anxiety was measured on day 8^th^ and on day 14^th^ using Elevated plus maze (EPM) and Vogel’s conflict test (VCT). After evaluating anxiety on day 14^th^, blood samples were collected for the estimation of serum lactate dehydrogenase (LDH). The animals were then sacrificed for MDA estimation in cardiac tissues and for the transmission electron microscopic (TEM) studies. DOX induced cardiomyopathy and exhibited anxiety-like effects in both EPM and VCT in mice. Pre and post treatment with Alprazolam provided dosedependent protective effect against DOX- induced cardiomyopathy in mice. Further, the drug improved (non-dose dependently) anxiety-like behaviour observed in cardiotoxic mice. Alprazolam appears to be a good candidate for treating anxiety associated with CV disorders for its observed antianxiety and cardioprotective effects in the present model.

**300**

**Exploring isoform specificity of nitric oxide synthase in ischemic postconditioning induced cerebroprotection in mice**

Kaur G, Kaur S, Singh N, Jaggi AS

Department of Pharmaceutical Sciences and Drug Research, Punjabi University, Patiala (Punjab), India.

The present study has been designed to investigate the isoform specific role of nitric oxide (NO) pathway in ischemic postconditioning (iPoCo) induced neuroprotection in mice. Bilateral carotid artery occlusion of 17 min followed by reperfusion for 24 h was employed to produce ischemia and reperfusion (I/R) induced cerebral injury in male swiss albino mice. Cerebral infarct size was measured using triphenyltetrazolium chloride staining. Memory was assessed using elevated plus maze test. Degree of motor incoordination was evaluated using inclined beam-walk test, rota rod test and lateral push test. Bilateral carotid artery occlusion followed by reperfusion, produced a significant rise in cerebral infarct size along with impairment of memory and motor coordination. Ischemic PoCo involving three episodes of 10 s carotid artery occlusion with intermittent reperfusion of 10 s produced a significant decrease in cerebral infarct size along with reversal of I/R induced impairment of memory and motor coordination. Ischemic PoCo induced neuroprotective effects were significantly abolished by pretreatment with N-nitro-l-arginine methyl ester, (L-NAME; 3 mg/ kg, i.p.); a non-selective nitric oxide synthase (NOS) inhibitor and N5-(1-iminoethyl)-l-ornithine, dihydrochloride (L-NIO, 30 mg/kg i.p.); a selective endothelial NOS inhibitor. However aminoguanidine, (AG, 400 mg/kg, i.p.); a selective inducible NOS inhibitor and 7-nitroindazole, (7-NI, 25 mg/kg i.p.); a selective neuronal NOS inhibitor, did not attenuate beneficial effects of iPoCo. It may be concluded that ischemic postconditioning induced neuroprotection involves nitric oxide pathway and this protection may further be mediated through endothelial nitric oxide mechanisms.

**301**

**To compare the short term safety and efficacy of citalopram and sertraline in depression**

Matreja PS^1^, Khosla PP^1^, Badyal DK ^2^, Deswal RS^2^

^1^Gian Sagar Medical College and Hospital, Ram Nagar, Banur, Patiala, ^2^Christian Medical College and Hospital, Ludhiana, India.

**Objective:**

Citalopram and sertraline are widely prescribed selective serotonin reuptake inhibitors (SSRIs). There is no conclusive evidence to show superiority of citalopram or sertraline in terms of efficacy or tolerability. Hence this study was designed to compare short term efficacy and safety of citalopram and sertraline in major depressive disorder (MDD) in Indian patients.

**Material and Methods:**

In an open, randomized study, 100 patients were divided into two groups. In Group A (*n* = 50) patients received citalopram (20-60 mg/day) for 6 weeks. In Group B (*n* = 50) patients received sertraline (50-150 mg/day) for 6 weeks. Patients were evaluated at baseline and then at 1, 2, 3, 4, 5, and 6 weeks.

**Results:**

There was significant improvement in Hamilton depression rating scale (HDRS), Montgomery and Asberg depression rating scale (MADRS) and Amritsar depressive inventory (ADI) scores (*P* < 0.05) with both the drugs. However, the decrease in score was more with citalopram (*P* < 0.05). Onset of action of citalopram was earlier as compared to sertraline (*P* < 0.05). The number of responders and remitters was also more with citalopram (*P* < 0.05). No serious adverse event was reported in either of the groups.

**Conclusion:**

Citalopram had shown better efficacy, earlier onset of action and more number of responders and remitters as compared to sertraline in MDD in Indian patients.

**302**

**Immunohistochemical localization of n-and l-type voltage-sensitive calcium ion channels in the spinal cord of morphine and morphine + nimodipine treated rats**

Ray S B, Verma D, Gupta YK

All India Institute of Medical Sciences, New Delhi, India.

Opioids like morphine produce antinociception by closing N- and P/Q-types of Voltage-sensitive calcium ion channels (VSCCs) expressed in presynaptic nerve terminals. However, continuous morphine administration leads to tolerance, which might be due to alterations in the VGCCs. It was hypothesized that co-administration of antagonists to the L-type VSCCs (at a low-dose) would increase the antinociceptive effect of morphine The study was conducted on Wistar rats (n=36), which were divided into 3 groups and administered the following drugs (1) Physiological Saline (2) Morphine (20 mg/kg for 7 days followed by 30 mg/kg for 7 days) and (3) Morphine+Nimodipine (morphine as in previous group + 2 mg/kg nimodipine once daily), for 14 days. Later, cryostat sections (20 µm thick) of spinal cords were processed for localization of N- (Anti-Ca_V_2.2 antibody; 1:100) and L-type (Anti-Ca_V_1.2 antibody; 1:200) VSCCs. Expression of these channels was quantified using image analysis software. Nimodipine increased the antinociceptive effect of morphine in the tail-flick and hot plate tests. Both L- and N-type VGCC were selected expressed in the superficial laminae (Laminae I-II) of the spinal cord in saline-treated group. Since spinal processing of pain also occurs in superficial laminae, this finding suggests an important role for these VGCCs. Further, chronic morphine administration significantly increased the expression of both L- and N-type VSCCs though nimodipine co-administration led to a decrease in L-type and an increase in N-type VSCC expression. The results indicate disordered Ca^2+^ influx into spinal neurons during tolerance, which is partially corrrected by nimodipine.

**303**

**Evaluation of nimodipine in cognitive impairment in albino mice**

Karande V, Jain S, Bende M

Govt. Med. College miraj, Maharashtra, India.

**Introduction:**

Nimodipine is a highly selective calcium channel blocker. Nimodipine has selective dihidropyridine receptors in limbic system. It has become apparent that in many cytotoxic events, Calcium homeostasis disruption occurs e.g. aging, stroke.

**Objective:**

To study the cognitive function enhancing effects of nimodipine by using Water maze test.

**Material and Methods:**

Albino mice of both sex were divided in three groups viz young control group (n=10), old control group (n=10) and nimodipine treated old mice (n=10). The first two groups were treated with normal saline (0.4. ml; I.P.) last group was treated with nimodipine (2 mg /kg I P); for 9 days. The albinos were tested on day 1 and day 5 and day 9 by using Water maze test. Vehicles and nimodipine were given half n hour before the test.

**Results:**

In water maze test the mice had to learn to search for the platform. Young mice were competent in learning and memory, right from the beginning and their performance further increased with training as evident by significant reduced latency to find the platform. and increased number of crossing of the platform area. In contrast, aged mice did not show significant performance on both two mentioned parameters. However daily treatment by nimodipine in another group of aged mice did show significant improvement in both the parameters of test on day 1 and after training on day 5 and day 9, but it did not reach the performance of young mice.

**Conclusion:**

The study suggest that nimodipine enhances the cognitive function in aged mice.

**304**

**Role of cytokines in diabetese-induced decrease of antinociceptive effect in mice**

Kumar R, Singh M

I.S.F. Institute of Pharmaceutical Sciences and Drug Research, Moga Punjab-142001, India.

**Introduction:**

Experimental diabetes mellitus is reported to attenuate antinociceptive effect in mice. Cytokines inhibitor/ antagonist is documented to restore the decreased antinociceptive effect of morphine in diabetic animals. Cytokines are known to induce the expression of inducible nitric oxide synthase (iNOS), and nitric oxide (NO) is reported to reduce the antinociceptive effect of morphine in diabetic mice. Furthermore, iNOS inhibitors are demonstrated to attenuate the tolerance developed to analgesic effect of morphine. Spleenectomy restores decrease antinociceptive effect of morphine in diabetic mice. Therefore, the present study has been designed to investigate the role of cytokines in diabetes-induced decrease antinociceptive effect of various analgesics.

**Materials and Methods:**

Streptozotocin (60 mg/kg *i.p.*, 4days) was administered to induce experimental diabetes in mice. Four weeks after administration of streptozotocin, the nociceptive threshold for morphine (10 mg/kg, *i.p.*), indomethacin (10mg/kg, i.p), and acetylsalicylic acid (400mg/kg, *i.p.*) in diabetic and non-diabetic mice was measured using tail-flick test (analgesiometer). Urinary / serum nitrite concentration was estimated using Greiss reagent. Spleen homogenate supernatant (SHS) was prepared from spleen of diabetic mice and administered in normal mice for15 days. Cyclosporine (25 mg/kg, *i.p,* 15 days) was administered to diabetic mice.

**Results:**

Analgesic effect of morphine, indomethacin and acetylsalicylic acid improved in splenectomised diabetic mice. Hyperalgesia was noted in both diabetic and SHS treated non-diabetic mice. Moreover, the levels of nitric oxide were also elevated in diabetic and SHS treated mice. Administration of Cyclosporine (25 mg/ kg, *i.p.*), an Il-2 inhibitor, attenuated diabetes and SHS induced decrease in nociceptive threshold and increase in serum and urinary nitrite levels.

**Conclusion:**

It may be concluded that an increase in cytokines release may be responsible for the observed decrease in antinociceptive effect of morphine, indomethacin and acetylsalicylic acid in diabetic mice.

**305**

**Possible sciatic nerve ligation induced behavioral, biochemical alterations in rats**

M Seema, K Anil, K Harikesh

Pharmacology Division, University Institute of Pharmaceutical Sciences, Panjab University, Chandigarh – 160014, India.

**Introduction:**

Chronic painful condition is very common problem now days. Present study was performed with aim to study the possible behavioral, biochemical alterations in sciatic nerve ligated rats.

**Materials and Methods:**

Sciatic nerves of male rats were ligated. Weight variation, behavioral (thermal hyperanalgesia, cold allodynia, anxiety like behavior). Behavioral assessments were made at weekly intervals for a period of three weeks. Biochemical alterations (lipid peroxidation, reduced glutathione, catalse and nitrite) were determined in both sciatic nerve and brain after behavioral assessments. However, surgery was performed but not ligated in na#x00EF;ve animals.

**Results:**

Sciatic nerve ligation significantly caused weight reduction, thermal hyperalgesia, and (hot plate test) and cold allodynia and anxiety like behavior in (plus maze test) after second and third week as compared to naïve animals (*P*<0.05. Biochemically, sciatic nerve ligated rat brain and nerve both significantly increased lipid peroxidation, nitrite concentration and depleted reduced glutathione and catalase activity (*P*<0.05). However, extent of oxidative damage was significantly higher in sciatic nerve as compared to rat brain.

**Conclusion:**

Sciatic nerve ligation significantly caused alteration in behavioral, biochemical parameters. Study further suggests that the sciatic nerve ligation is a reliable method to study chronic painful condition and their related problems.

**306**

**Fenofibrate ameliorates ICV streptozotocin induced cognitive deficits and oxidative stress**

Gangwani S, Sharma V, Deshmukh R, Bedi KL

I.S. F. College of Pharmacy, Moga, Punjab-142001, India.

**Objective:**

Present study was designed to evaluate the role of Fenofibrate (PPAR agonist) in cognitive dysfunction and oxidative stress induced by intracerebroventricular (ICV) Streptozotocin (STZ) in rats.

**Materials and Methods:**

Present study was conducted on male wistar rats (250-280g). Intracerebroventricular streptozotocin(3mg/kg) was given bilaterally on days 1 and 3 to induce experimental dementia resembling Alzheimer’s type. The rats were treated chronically with Fenofibrate (50, 100 and 200mg/kg, ip) for a period of 21days starting from 1^st^ day of STZ administration. Learning and memory was assessed using Morris water maze and Modified elevated plus maze task paradigms. Locomotor activity was examined with Actophotometer. Brain acetylcholinestrase activity was measured by Ellman method. Malonialdehyde, Nitrite and Glutathione levels were measured by Wills *et al*., Ellman *et al*., and Green *et al*., respectively. Lactate dehydrogenase was estimated with commercially available kit and the total protein content was measured by Biuret method. Activity of test drug against neuroinflammtion was examined by assessing levels of TNFα and IL6 using ELISA and flow cytometry.

**Results:**

ICV STZ treated rats exhibited poor retention of memory in Morris water maze and Modified elevated plus maze task paradigms. and showed increased levels TNF alpha and IL6, oxidative–nitosative stress and cholinergic impairment. Chronic treatment with Fenofibrate dose dependently reversed all the changes induced by ICV STZ.

**Conclusion:**

Neuroprotection offered by PPAR alpha agonist in Intracerebroventricular streptozotocin induced dementia of Alzheimer’s type may be due to its anti-inflammatory and its antioxidant activity.

**307**

**Evaluation of ethanolic extract of *Vitex negundo* leaves on anxiolytic activity in mice**

Barman S, Das S

Assam Medical College, Dibrugarh, India.

**Introduction:**

About 15-20% of the population suffers from anxiety disorders and newer antianxiety drugs are being evaluated to overcome this problem.*Vitex negundo* belongs to the Verbenaceae family. The leaves of this plant are astringent, febrifuge, sedative, tonic and vermifuge and used in acute rheumatism, gonorrhoea and ulcers. It has also antibacterial, antitumour, insecticidal and fumigant activity.

**Methods:**

Healthy swiss albino mice (20-25g) of either sex were divided into four groups of six animals each.

Group A –Normal control; 3% gum acacia
Group B – Standard control; diazepam (2mg/kg, i.p.)
Group C – test group; EVNL (40mg/kg, i.p.)
Group D – test group; EVNL (60mg/kg, i.p.)

In both the experimental models the test and standard drug were administered 30 minutes before the animals were exposed to the respective models of anxiety viz., elevated plus maze (EPM) and mirrored chamber (MC) models.

**Results:**

In EPM model there was a significant (*P*<0.01) increase in percent entries in open arm and increased number of entries and time spent on open arms (*P*<0.01) in group B, C and D as compared to group A. In MC model there was a significant (*P*<0.01) decrease in latency to enter the chamber and a significant (*P*<0.01) increase in number of entries and time spent in the chamber in group B, C and D as compared to group A. The observations were noted in 5 minute session.

**Conclusions:**

As revealed by the above study ethanolic extract of *Vitex negundo* leaves possesses significant anxiolytic activity.

**308**

**Pioglitazone induced beneficial effects in experimental dementia: An evidence of its antiinflammatory action**

Rinwa P, Kaur B, Singh N, Jaggi AS

Department of Pharmaceutical Sciences and Drug Research, Punjabi University, Patiala (Punjab), India.

Neuroinflammation is known to play a key role in the pathogenesis of CNS disorders like AD. The present study was undertaken to investigate possible mechanism of Pioglitazone induced beneficial effect in memory deficits associated with experimental dementia. Dementia was induced in swiss albino mice by administration of streptozotocin (3 mg kg^-1^ administered intracerebroventricularly on 1^st^ and 3^rd^ day). Morris water- maze test was employed to assess learning and memory of the animals. Brain myeloperoxidase (MPO) levels and neutrophilic infiltration were assessed as an index of neuroinflammation. Blood glucose level was also measured. Streptozotocin (STZ) produced a significant decrease in water maze performance of mice hence reflecting loss of learning and memory. Pioglitazone (20 mg/kg p.o. daily for 15 days) successfully attenuated STZ induced memory deficits, without any significant per se effect on blood glucose levels. Higher levels of brain MPO activity and neutrophil infiltration was observed in STZ treated animals, which were significantly attenuated by Pioglitazone treatment. It is concluded that anti-dementic effect of Pioglitazone involves its potential anti-inflammatory actions.

**309**

**Anticonvulsant activity of *Citrus maximus* (pomelo) leaves in experimental animal models**

Thakuria N, Gohain K, Das S

Assam Medical College and Hospital, Dibrugarh, India.

**Introduction:**

Epilepsy refers to a disorder of brain function characterized by the periodic and unpredictable occurrence of seizures, whereas seizure means a transient alteration of behaviour due to disordered, synchronous, and rhythmic firing of populations of brain neurons. *Citrus maximus* (pomelo) is the biggest of all citrus fruits; each fruit weighing from 0.5-1.4 kg and belongs to the family Rutaceae of the kingdom Plantae. Traditionally, various parts of the plant like the leaves, flowers, fruit pulp and the rind are being used in cough, dyspepsia, as stomachic, in urinary disorders and insomnia.

**Methods:**

Healthy Wistar albino rats (*Rattus norvegicus*) weighing 100-150 gm of either sex were taken and divided into five groups having six animals in each group and the anticonvulsant activity for the ethanolic extract of *Citrus maximus* leaves (EECM) was evaluated by Maximal Electroshock (MES) method and Pentylenetetrazol (PTZ) induced seizure models. In both the models, there were a control (3% gum acacia), standard (Phenytoin for MES and Diazepam for PTZ), and three test-groups of 50, 100 and 200 mg/kg doses (selected after acute toxicity testing) and all the drugs were administered intraperitoneally.

**Results:**

EECM produced a dose dependent anticonvulsant activity in both MES (by reducing the extensor phase of convulsions) and PTZ (by delaying the onset of convulsions) seizure models which was found to be highly significant (*P*<0.01) with the optimal effect being observed at 200 mg/kg dose. Conclusions: The study demonstrated the potential anticonvulsant activity of *Citrus maximus* leaves for both grand-mal and petit-mal epilepsy.

**310**

**Effect of ketamine on seizure activity and its interactions with antiepileptic drugs in rats**

Dashputra AV, Borkar AS, Hemnani TJ, Badwaik RT

NKP Salve Institute of Medical Sciences and Research Centre, Nagpur, Maharashtra, India.

**Introduction:**

There is controversy regarding use of Ketamine as general anesthetic agent in patients of epilepsy and both pro and antiepileptic effect has been documented in clinical practice. It has been shown that Ketamine minimizes seizureinduced brain damage and its combination with antiepileptic drugs also prevents degeneration of thalamic neurons induced by focal cortical seizures. It was therefore decided to explore the effect of ketamine on seizure activity and its interactions with antiepileptic drugs in rats. To study the effect of Ketamine on seizure activity and its interactions with antiepileptic drugs in rats.

**Materials and Methods:**

Male albino rats were taken for study of antiepileptic activity of drugs 30 minutes after their intraperitoneal administration by methods of supramaximal electroshock seizures and chemoshock (pentylenetetrazol) seizures.

**Results:**

Ketamine showed protection against electroshock seizures whereas, ketamine enhanced chemoshock seizures. Combined treatment of ketamine with antiepileptic drugs exerted a much stronger protective effect against electroshock seizures than either drug alone which was significant when ketamine was combined with sodium valproate and highly significant when ketamine was combined with phenytoin and fosphenytoin. In chemoshock seizures ketamine showed protection in combination with benzodiazepines, antagonism with phenytoin and fosphenytoin while no change was noticed in combination with sodium valproate and phenobarbitone.

**Conclusion:**

In electroshock method ketamine proved to be antiepileptic acting synergistically with sodium valproate, phenytoin and fosphenytoin. In chemoshock method ketamine acts as proconvulsant and variably modulates the action of other antiepileptic drugs.

**311**

**5-HT_2a_ receptor binding and antidepressant studies on Anximin^®^, a polyherbal formulation**

Mishra S^1^, Khanna VK^2^, Kumar V^1^

^1^Neuropharmacology Laboratory, Department of Pharmaceutics, Institute of Technology, Banaras Hindu University, Varanasi 221 005, Uttar Pradesh, ^2^Developmental Toxicology Division, Indian Institute of Toxicology Research, Lucknow 226 001, Uttar Pradesh, India.

**Introduction:**

The objective of present study was to evaluate the antidepressant activity of Anximin. It composed of five medicinal plants’ extracts namely *Bacopa monnierae, Convolvulus pluricaulis, Rauwolfia serpentina, Nardostachys jatamansi and Acorus calamus..*

**Methods:**

Adult Charles foster rats (200±20g) and Wistar mice (25±5g), of either sex were used. Antidepressant activity was assessed by using the validated models of depression viz. behavioural despair test (BDT), tail suspension test (TST), learned helplessness test (LHT) and yohimbine toxicity enhancement test (YTE). Anximin (20 and 40 mg/kg) was given orally for 7 consecutive days as a suspension in 0.3% CMC. Imipramine (10 mg/kg, p.o.) was used as standard antidepressant drug. All the behavioural experiments were performed 1 hr after last administration of drug on day 7. To elucidate mechanism of action, a receptor binding study was also performed using rat’s frontal cortex.

**Results and Conclusion:**

Immobility time in FST and TST was significantly (*P* < 0.01) reduced by the high dose of Anximin, while low dose has no significant effect. A significant (*P* < 0.001) decrease in number of escape failures in LHT was also observed in both groups of Anximin treatment. In the YTE test, the percent mortality of animals was 33% and 66% (for 20 and 40 mg/kg respectively) whereas with imipramine treated animals mortality was 100%. The high dose of Anximin significantly (*P* < 0.05) decreased the binding level of 3[H] ketanserin, indicating a downregulation of 5-HT_2A_ receptors. The results indicate that Anximin possesses promising antidepressant activity acting through 5-HT_2A_ receptors.

**312**

**Baicalein an antioxidant and lipoxygenase inhibitor reverses cognitive dysfunction and oxidative stress in experimental model of Alzheimer’s disease**

Sharma N, Bansal V, Sharma V, Deshmukh R, Bedi KL

I.S. F. College of Pharmacy, Moga, Punjab-142001, India.

**Objective:**

To evaluate the neuroprotective efficacy of baicalein in cognitive dysfunction and oxidative stress induced by intracerebroventricular (ICV) colchicine in rats.

**Materials and Methods:**

Colchocine (COL) (15*µ*g/5*µ*l) was given intracerebroventrically (ICV) to induce experimental dementia of Alzheimer’s type in male wistar rats(260-300g). The rats were treated chronically with Baicalein (1, 2 and4 mg/kg) for a period of 21 days. Learning and memory was assessed with Morris water maze and Modified elevated plus maze task paradigms. Locomotor activity was examined with photoactometer. Brain acetylcholinestrase activity was measured by Ellman method. Malonialdehyde (MDA), Ntrite and Glutathione (GSH) levels were measured by Wills et al., Ellman et al., and Green at al., respectively. Lactate dehydrogenase (LDH) was estimated with commercially available kit and the total protein content was measured by Biuret method.

**Results:**

ICV COL treated rats exhibited poor retention of memory in Morris water maze and Modified elevated plus maze task paradigms. Chronic treatment with Baicalein, dose dependently improved Learning and memory impairment and showed antioxidant activity by significantly reducing elevated levels of Malondialdehyde (MDA), nitrite and restoring depleted levels of glutathione. It decreased elevated levels of AChE and LDH confirming its effect on neurotransmission and cell viability.

**Conclusion:**

ICV COL in rats mimics changes observed in Alzheimer kind of dementia i.e impaired cholinergic neurotransmission, cerebral glucose and energy metabolism and the subsequent oxidative-nitrosative stress. Results of present study clearly indicate that systemic administration of Baicalein has a neuroprotective role and it shows lot of promise as a future drug candidate in finding out a cure for Alzheimer’s treatment

**313**

**The GRIK3 SER310ALA polymorphism is associated with schizophrenia in Indian population**

Ahmad Y^1^, Bhatia MS^1^, Mediratta PK^1^, Sharma KK^1^, Sinha S^2^

^1^University College of Medical Sciences, Guru Teg Bahadur Hospital, Shahdara-110095, ^2^All India Institute of Medical Sciences, Ansari Nagar, New Delhi-110029, India.

Schizophrenia is a severe psychiatric illness characterized by disturbance of thought, hallucination and delusions. Several studies have suggested that dysfunctions in the glutamatergic transmission are linked to the pathogenesis of schizophrenia. The excessive activation of glutamate receptors seems to be related to the disruption of neuronal ionic gradients leading to excitotoxicity with primarily involvement of kainate ionotropic glutamate receptors in this mechanism. The GRIK3 gene encoding for the ionotropic glutamate receptor kainate 3 contains a functional polymorphism (T928G) leading to the substitution of a serine with an alanine in position 310 of the protein sequence. The T928G (Ser310Ala) polymorphism of ionotropic glutamate receptor kainate 3 gene (GRIK3) and its positive association with schizophrenia was reported in Caucasians, whereas, no association of this polymorphism with schizophrenia was shown in two other populations, Chinese and Japanese. The genetic susceptibility profiles of Indians are different in many respects from those of white Caucasians, Chinese or Japanese. We performed an association study between the Ser310Ala GRIK3 polymorphism and schizophrenia in a sample of 100 schizophrenic patients and 100 controls and 50 patient controls (neuropsychiatric patients other than schizophrenics) in Indian population by PCR-RFLP. We found a statistically significant difference in the genotype and allelic distributions (*P*<0.000001 and *P*=0.01 respectively) of Ser310Ala polymorphism in schizophrenics as compare to healthy control and in particular considering the ala allele as dominant (Odds ratio, OR=1.7, 95% of confidence interval, CI=1.137-2.540). This finding suggests a potential role for GRIK3 for susceptibility to schizophrenia in Indian population.

**314**

**Central nervous system depressant activity of *Sida acuta* root extract in mice**

Bansod MS, Rajgure DT, Harle UN, Vyawahare NS, Yende SR

Aissms college of pharmacy, Pune, India.

Neuropharmacological evaluation of hydroalcoholic root extract of *Sida acuta* (Malvceae) was studied in Swiss albino mice. General behavior, locomotor activity, muscle relaxant activity, phenobarbitone sodium-induced sleeping time were studied using standard paradigms’. The results revealed that *Sida acuta* (SA) root extract showed significant reduction in spontaneous activity (general behavioral profile) during blind screening, significant reduction in locomotor activity evaluated by actophotometer and significantly potentiation of phenobarbitone sodium-induced sleeping which indicate general depression of central nervous system. However, the SA extract did not produce any impairment in muscle relaxant activity (rota-rod test), which indicate that SA does not act on skeletal muscles. The phytochemical study of SA extract revealed the presence of alkaloids, flavonoids, tannin and sterol. The results suggest that the hydroalcoholic extract of *Sida acuta* shows general central nervous system inhibitory action and becomes a candidate for further pharmacological evaluation.

**315**

**Anti-depressant and analgesic like activity of 5-HT_3_ antagonist - novel approach in treating co-morbid depression and pain**

Viyogi S, Mahesh R, Pandey D, Rajkumar

Birla Institute of Technology and Science, Pilani, Rajasthan, India.

Chronic pain and depressive illnesses are debilitating disease states that are not only associated with each other but also are variably resistant to currently available therapeutic agents. Serotonergic neurons have a regional distribution in brain areas implicated in a range of neurological phenomenon and there has been much interest in the therapeutic potential of 5-HT_3_ receptor antagonists for anti-depressant, antinociceptive and other psychiatric disorders. The behavioral investigation for depression related symptom involves rodents test battery like, olfactory bulbectomy (OBX) showing increased hyperactivity, increased fecal pellet and rearing in open filed test, improved immobility in forced swim test and tail suspension test, and for pain (Formalin and acetic acid induced pain, tail flick method). The acute treatment of 5-HT_3_ antagonist Ondansetron (OND) (0.5-2mg/kg), i.p. significantly decreased the flinching and writhing induced in formalin and acidic acid respectively. Latency in tail flick method was significantly increased with OND treatment. Chronic treatment with Ondansetron restored the behavioral dysfunction in OBX rats. Above preliminary studies showed the analgesics and anti-depressant like effects of 5HT_3_ antagonist. The present finding indicates the peripheral and central activity of OND in depression and pain. Our study strengthens our hypothesis highlighting the potential role of Ondansetron as possible novel target for chronic comorbid pain and depression. Still prospective work is going on to evaluate the role of 5-HT_3_ antagonist in this field.

**316**

**Homocysteine levels in neurological disorders in Indian population**

Sharma S, Prakash A, Kushwaha S

Institute of Human Behavior and Allied Sciences, Delhi, India.

Hyperhomocysteinemia is an important risk factor for stroke and other neurological disorders and there is paucity of data in Indian population. The main objective was to study the relationship between homocysteine (Hcy) levels and neurological disorders namely – stroke, dementia and epilepsy. A retrospective analysis of 418 serum Hcy level collected over last 3 years during the period 2004 –2007 was done. Out of 418 requisitions, there were 55% cases of stroke, 15.33% dementia, 4.87% epilepsy, 2.67% behavior disorder, 1.21% homocysteniuria and 20.9% others. Mean plasma Hcy levels (*µ*mol/l) in patients with stroke was 18.92±0.75, dementia 18.43±1.36, epilepsy 24.93±2.93, behavior disorder 15.93±1.85, homocysteniuria 35.19±7.09 and 17.41±1.93 in miscellaneous group. Coexisting diseases in decreasing order were: hypertension (31.3%) > renal failure (21%) > DM (6.2%) > CAD (2.3%) > cranial nerve palsy (2.1%) > hyperlipidemia (1.4%). Hcy levels were significantly higher in patients with dementia and epilepsy (*P*<0.05). Even though not statistically significant but higher Hcy levels were observed in hypertension > renal failure > coffee and alcohol intake. Hcy levels were significantly higher in the age group of 31-40 y and highest in patient >70 y of age (predominantly males) with mean plasma Hcy level of 22.75±1.83 *µ*mol/l. Mean Hcy levels in patients taking coffee and alcohol was 20.3±1.38 *µ*mol/l. The present study shows homocysteine level is an important risk factor in dementia and epilepsy patients and increases with age. Further the effect of Antiepileptic drugs on homocysteine should be studied.

**317**

**Protective effect of ibuprofen and alpha-lipoic acid on colchicine- induced model of Alzheimer’s disease in rats**

Mediratta PK, Khurana S, Banerjee BD, Sharma KK

University College of Medical Sciences, and GTB Hospital, Delhi, India.

**Introduction:**

Neuroinflammation and oxygen free radicals (OFR) reportedly play an important role in pathophysiology of Alzheimer’s disease (AD). The aim of the present study was to evaluate the effect of ibuprofen, an anti-inflammatory agent and alpha-lipoic acid, an antioxidant on colchicine induced model of AD.

**Methods:**

Male Wistar rats (250 – 350 g) were used. Ibuprofen (20 and 40mg/kg orally (p.o) twice daily) and alpha-lipoic acid(100 and 200 mg/kg p.o./day) were administered one week prior to colchicine (15 ug/5 ul i.c.v.) and three weeks after it. Rivastigmine (2.5 mg/kg p.o. /day) was used as a standard drug. The learning and memory behavior were assessed using elevated plus maze, step down latency and Morris water maze on days 13, 14 and 21. The parameters of oxidative stress were assessed by measuring the malondialdehyde (MDA), and reduced glutathione levels in brain tissue on day 22 of the colchicine injection.

**Results:**

Central administration of colchicine produced cognitive dysfunction, increased MDA levels and decreased reduced glutathione levels. Chronic Treatment with ibuprofen and alpha-lipoic acid significantly improved colchicine - induced cognitive impairment. Further, they also significantly reduced lipid peroxidation and restored reduced glutathione levels.

**Conclusion:**

Inflammation and oxidative stress appears to play a role in cognitive dysfunction in animal models of AD as evidenced by antagonism of colchicine-induced impairment of cognitive dysfunction and oxidative stress by ibuprofen and alpha-lipoic acid. The results of the present study indicate that ibuprofen and alphalipoic acid treatment has a neuroprotective role against colchicine - induced model of AD in rats.

**318**

**Neuroprotection by curcumin and piracetam on colchicine-induced cognitive dysfunction and oxidative stress in rats**

Khurana S, Mediratta PK, Banerjee BD, Sharma KK

University College of Medical Sciences, and GTB Hospital, Delhi, India.

**Introduction:**

Oxidative Stress and neuroinflammation play a significant role in pathophysiology of Alzheimer’s disease (AD). The present study was aimed to evaluate the effect of curcumin and piracetam against colchicine – induced (a model of AD) cognitive dysfunction and oxidative stress in rats.

**Methods:**

Male Wistar rats (250 – 350 g) were used in the study. Curcumin (200 and 400mg/ kg orally (p.o)/day) and piracetam (400 and 800 mg/kg p.o. /day) were administered one week prior to colchicine (15 ug/5 ul i.c.v.) and three weeks after it. Rivastigmine (2.5 mg/kg p.o. /day) was used as a standard drug. The learning and memory behavior were assessed using elevated plus maze, step down latency and Morris water maze on days 13, 14 and 21. The parameters of oxidative stress were assessed by measuring the malondialdehyde (MDA), and reduced glutathione levels in brain tissue on day 22 of the colchicine injection.

**Results:**

Central administration of colchicine produced cognitive dysfunction, increased MDA levels and decreased reduced glutathione levels. Chronic Treatment with curcumin (200 and 400 mg/kg daily) and piracetam (400 and 800mg/kg daily) for a period of 28 days beginning 7 days prior to colchicine injection significantly improved colchicine - induced cognitive impairment. Further, they also significantly reduced lipid per oxidation and restored reduced glutathione levels.

**Conclusion:**

There is evidence for increase in free radical generation and subsequent oxidative stress leading to cognitive impairment in animal models of AD which is antagonized by both curcumin and piracetam. The results of the present study indicate that curcumin and piracetam treatment has a neuroprotective role against colchicine - induced cognitive impairment and associated oxidative stress.

**319**

**Six interesting case reports of neuropsychiatric adverse drug reactions of commonly prescribed drugs in a tertiary care hospital**

Afonso N, Dang A, Rataboli PV

Goa Medical College, Goa, India.

**Background:**

Clinicians prescribe various drugs for treating diseases. Many of these drugs can cause important neuropsychiatric Adverse Drug Reactions (ADRs) that the physicians are unaware of. They often mimic a clinical entity.

**Aim:**

We aim to highlight some of the neuropsychiatric ADRs, which occured with commonly prescribed drugs, in a tertiary care hospital, over a period of 6 months.

**Materials and Methods:**

Doctors and interns voluntarily reported neuropsychiatric ADRs occurring in different wards of the tertiary care hospital, using “Drop Boxes” and “ADR reporting forms”, over a period of 6 months. Information uncovered was interpreted in light of related psychiatric literature.

**Results:**

6 neuropsychiatric ADRs were reported over 6 months. Nightmares and psychosis were seen in 2 patients each. One patient each, presented with hallucinations and sleep eating disorder. The age group of 20-40 years was the most vulnerable. Males were more affected than females. Among the drugs, Flouroquinolones were implicated in 3 of the cases, followed by Zolpidem (2) and Mirtazepine (1). Causality assessment by Naranjo Algorithm indicated that 5 of the cases were “Probable”. One case, receiving ciprofloxacin, showed a “moderate” neuropsychiatric complication (according to “Modified Hartwig and Seigel” scale) and needed hospitalisation on this account.

**Conclusion:**

In case of neuropsychiatric ADRs the clinician should consider immediate discontinuation of the causative medication. Safety can be augmented by an effective ADR reporting system with supportive access to basic and specialized mental health services. Doctors should think of drugs as a causative factor before diagnosing a psychiatric condition.

**320**

**Nose-to-brain administration of a radiolabeled cholinesterase inhibitor in a rat model for neurodegenerative disorders**

Bhavna^1^, Ali M^1^, Bhatnagar^2^ A, Ali J^1^

^1^Department of Pharmaceutics, Faculty of Pharmacy, Jamia, Hamdard, New Delhi; ^2^Department of Nuclear Medicine, Institute of Nuclear Medicine and Allied Sciences, Delhi, India.

**Introduction:**

Earlier studies have demonstrated the use of intranasal route as an alternative route of administration for rapid drug delivery to the brain as it is a practical and noninvasive route. The objective of this investigation was to prepare nanoparticulate nasal formulation of a cholinesterase inhibitor. The chitosan solution was taken as a carrier via nose-to-brain delivery. A further objective was to characterize and evaluate the formulation on the basis of particle size, particle size distribution and *in vivo* evaluation using pharmacoscintigraphy.

**Methods:**

The chitosan nanoparticles were prepared by ionic gelation method. The formulation of chitosan nanoparticles of cholinesterase inhibitor was radiolabeled using technetium-99 (Tc_99m_) by drylabeling method. The radiolabeling efficiency was determined by ascending instant thin layer chromatography (TLC). The optimized, stable radiolabeled-drug formulations of cholinesterase inhibitor were used for biodistribution study in rats.

**Results and Conclusion:**

Two nasal formulation viz the drug solution and the nanoparticulate drug formulation were compared. The Swiss albino rats (male, aged 4–5 months) weighing between 200 to 250 g were selected for the study. Radiolabeled drug formulation Tc_99m_ was administered in each nostril by canula (size 18/20). The rats were anaesthetized using 0.25 ml diazepam intramuscular injection (10mg/ml) and placed on the imaging board. Imaging was performed using Single Photon Emission Computerized Tomography (SPECT), provided by GE healthcare system and the images were recorded using the software Entegra version. The experiment for radiometery analysis was also performed on Swiss albino rats weighing between 200 and 250 g. Three rats for each formulation per time point was used in the study. Radiolabeled drug formulation were instilled into the nostrils with the help canula of (size 18/20). The rats were killed humanely at different time intervals and the blood was collected using cardiac puncture. Subsequently, brain and other tissues (liver, spleen, intestine, kidney and tail) were dissected, washed twice using normal saline, made free from adhering tissue/fluid, and weighed. Radioactivity present in each tissue/organ was measured using shielded welltype gamma scintillation counter. Radiopharmaceutical uptake per gram in each tissue/organ was calculated. These studies showed that chitosan nanoparticulate were retaining for a prolonged period and circumvented the BBB.

**321**

**Losartan and propranolol annul hypertensive effect but potentiate anti-depressant action of venlafaxine in Wistar rats and Swiss mice**

V Vivek, Patil PA

JN Medical College, Belgaum, India.

**Introduction:**

Venlafaxine, commonly used for treatment of depression is known to increase blood pressure (BP) depending on its dose and duration of exposure, intending the use of an antihypertensive. Since its anti-depressant activity is partly through norepinephrine reuptake inhibition, sympatholytic anti-hypertensives coadministered to correct hypertension, could be expected to modify its pharmacological action. Also anti-hypertensive like losartan, an angiotensin-antagonist could influence its anti-depressant action, as it is been reported to possess anti-depressant activity. As there is scanty information of interaction of venlafaxine with prazosin, propranolol and losartan, the present study was planned to investigate the same in male Wistar rats and Swiss mice.

**Methods:**

Healthy adult male Wistar rats and Swiss mice weighing 150±25g, 25±5g respectively were acclimatized under 12; 12 light-dark cycle for a week. Anti depressant activity was assessed by Forced swim test (FST), Tail suspension test (TST). Rat and mice dose were calculated from human therapeutic equivalent dose, sub effective dose (SED) for each drug were established in different experimentals for their psychotropic activity and interaction study was run for 30 days. All the groups were screened for psychotropic activity and BP on 0, 15 and 30^th^ day.

**Results:**

SEDs of propranolol and losartan combined with SED of venlafaxine significantly (*P*<0.001, *P*<0.01) reduced immobility time almost to that of venlafaxine in therapeutic dose in both FST and TST. (fig 1).

**Conclusion:**

Losartan and propranolol but not prazosin may be useful to lower BP increased by venlafaxine therapy and may decrease other adverse drug reactions of venlafaxine by decreasing its dose.

**322**

**Neuropharmacological effects of *Ocimum sanctum***

Chatterjee M, Palit G

Central Drug Research Institute, Lucknow, India.

*Ocimum sanctum* (OS), a medicinal plant, is extensively used in the Indian system of medicine for a variety of clinical disorders. The ethanolic extract of OS was investigated for its anti-anxiety and antidepressant effects in various animal models in mice. Graded doses (25-200mg/kg p.o. body weight) of OS showed dose dependent CNS depressant activity as evaluated using Digiscan animal activity monitor and a diminution in the anxiety response as observed against elevated plus maze, light dark and hole board tests, which signifies its anti anxiety activity. Further at a dose of 50mg/kg p.o. body weight, the OS extract markedly shortened the immobility time in the forced swimming test (FST) and tail suspension test (TST), indicating a possible antidepressant activity and the effect were compared with the respective standard drugs. However, at higher doses of 200mg/kg p.o., the extract was found to be sedative in nature but it did not affect the motor coordination of the mice in the rotarod test. These results show that the OS extract has significant neuropharmacological activity and provides support for its potential anxiolytic and antidepressant activity.

**323**

**Effects of trimetazidine on electrically and chemically - induced convulsions, anxiety and locomotor behaviour in mice**

Jain S, Bharal N, Mediratta PK, Sharma KK

University College of Medical Sciences and GTB Hospital, Delhi- 110095, India.

**Introduction:**

Trimetazidine (TMZ) is an effective and well tolerated anti-ischemic agent. It has demonstrated antioxidant, antinociceptive, and anti inflammatory activity in various experimental models, whilst experimental data on its effect on neurobehavior are essentially lacking. The present study was designed to investigate the effects of TMZ on electrically and chemically –induced convulsions, anxiety and locomotor activity in mice.

**Methods:**

In the present study, TMZ was administered in doses, 5, 10 and 20 mg/kg (single dose, orally) in healthy Swiss albino mice (24-30 g). Phenytoin (12.5 and 25 mg/kg, orally) was used as standard anticonvulsant drug. The increasing current electroshock (ICES) and pentylenetetrazole (PTZ)- induced convulsions tests were used to assess anticonvulsant activity while elevated plus maze and actophotometer tests were performed to observe its effect on anxiety and locomotor activity, respectively.

**Results:**

TMZ in 10 and 20 mg/kg doses significantly raised the seizure threshold current in ICES test. Co-administration of sub-threshold dose of TMZ (5mg/kg) with sub-threshold dose of phenytoin (12.5 mg/kg) caused significant increase in seizure threshold current. In PTZ test, TMZ (20 mg/kg) significantly prolonged the latency to myoclonic jerk and generalized seizures in PTZ treated animals. No effect was observed on anxiety and locomotor activity in the studied animals.

**Conclusion:**

The anticonvulsant effect of TMZ could be due to its modulatory effect on calcium or sodium channels. Furthermore, it also possesses antioxidant property which may contribute to its anticonvulsant activity. Thus TMZ appears to possess anticonvulsant activity and is devoid of anxiogenic effect in mice.

**324**

**Neuropeptide Y Y1 receptors in the central nucleus of amygdala mediate the anxiolytic-like effect of progesterone in mice: Behavioral and immunocytochemical evidences**

Kokare DM, Deo GS, Dandekar MP, Subhedar NK

Department of Pharmaceutical Sciences, RTM Nagpur University, Campus, Amravati Road, Nagpur-440 033, India.

**Objective:**

Previous study reported that progesterone (PROG) and its metabolite allopregnanolone (ALLO) increases neuropeptide Y (NPY) Y1 receptors gene expression in the amygdala. Herein, we examined the involvement of NPYY1 receptors in the actions of PROG/ALLO employing behavior and immunocytochemistry as tools of analysis.

**Materials and Methods:**

NPY, NPYY1 receptors agonist [Leu^31^, Pro^34^]-NPY or antagonist BIBP3226 was administered bilaterally into the central nucleus of amygdala (CeA), alone or in combinations with PROG (ip), to adult male mice. Anxiety-like behavior was evaluated in terms of number of licks and shocks in the Vogel conflict test (VCT). The effect of acute ALLO (ip) on the endogenous NPY system in the CeA, ventral part of lateral division of bed nucleus of the stria terminalis (BSTLV), nucleus accumbens core (AcbC) and arcuate nucleus (ARC) was studied with immunocytochemistry.

**Results:**

Intra-CeA administration of NPY or [Leu^31^, Pro^34^]-NPY and PROG (ip) produced dose dependent increase in number of licks and shocks in VCT, suggesting anxiolyticlike effect; BIBP3226 treatment caused opposite effects. While prior administration with NPY and [Leu^31^, Pro^34^]-NPY at subeffective doses potentiated the PROG/ALLO induced anxiolytic-like effect, BIBP3226 antagonized the same. Acute ALLO treatment dramatically decreased the population of NPY-immunoreactive cells in the CeA and ARC. While moderate increase was noticed in NPYimmunoreactive fibers in the AcbC and BSTLV, cells in AcbC and fibers in ARC showed no change.

**Conclusion:**

NPY might mediate the PROG induced anxiolytic-like behavior in the neuroanatomical framework of the CeA, possibly via NPYY1 receptors.

**325**

**Discovery of isatinimino derivatives as new leads for neuropathic pain treatment**

Semwal A., Ragavendran J. V., Dharmarajan S., Perumal, Yogeeswari

Birla Institute of Technology and Science, Pilani, India.

Neuropathic pain is defined as pain initiated or caused by a primary lesion, injury or dysfunction in the central or peripheral nervous system and is an area of largely unmet therapeutic need. At present, there are only few effective and well tolerated therapies available for neuropathic pain. Gama-aminobutyric acid (GABA) and Isatin have been implicated in pharmacotherapy of different neurological disorders including pain. In the present study, we have evaluated 10 novel isatinimino GABA derivatives (ARV1-10) for peripheral analgesic activity in acetic acid induced writhing in mice and antiallodynic and antihyperalgesic activities in chronic constriction nerve injury (CCI) and spinal nerve ligation injury model (SNL) in rats. Behavioral signs of different components of neuropathic pain namely; spontaneous pain, dynamic allodynia, cold allodynia and mechanical hyperalgesia were measured on day 9 post-surgery. All the compounds were administered intraperitoneally at the dose of 100 mg/kg. In acetic acid induced writhing response, ARV-4, ARV-5 and ARV-9 were the most active compounds with more than 80% inhibition. In CCI model, compound ARV-9 emerged as the most active compound, being effective in three out of four behavioral tests and compounds ARV-1, ARV-2, ARV-6 and ARV-7 were active in two behavioral assays. In SNL model, compounds ARV-3, ARV-7 and ARV-9 had shown complete protection in all the four behavioral studies and compounds ARV-4 and ARV-6 exhibited activity in three of the four nociceptive assays. Compounds ARV-1 and ARV-8 were observed to be active in two of the behavioral tests.

**326**

**Anticonvulsant activity of alcoholic extract of stems of *Artocarpus heterophyllus* (moraceae) in mice**

Jain AP, Tote MV, Undale VR, Sonawane SA

S.G.R.S College of Pharmacy, Saswad, Pune-412301, India.

The objective of the present study was to assess the anticonvulsant activity of alcoholic extract of stems of *Artocarpus heterophyllus* Lam (AEAH) in mice. The plant has been used in the treatment of diseases related to the nervous system as an antiepileptic drug in traditional medicines. Artocarpine, artocarpesine, artocarpetin, artocarpetin A, cycloheterophyllin and artonins A and B are prenylflavonoids present in *Artocarpus heterophyllus* Lam. Preliminary phytochemical studies revealed the presence carbohydrates, flavonoids, tannins, saponins and triterpenoids in AEAH. Anticonvulsant activity was evaluated in pentylenetetrazole (PTZ), strychnine and maximal electroshock (MES) induced convulsion models. Pretreatment with AEAH (250 and 500 mg/kg, po) significantly delayed the onset of PTZ and strychnine induced clonic convulsions and duration of extensor phase in MES induced convulsions indicating that this extract possess anticonvulsant activity. These results suggest that at doses 250 and 500mg/kg AEAH, exhibited anticonvulsant activities.

**327**

**Experimental studies on the anti-oxidant role of nitric oxide during stress**

Gulati K^1^, Chakraborti A^1^, Banerji BD^2^, Ray A^1^

^1^Vallabhbhai Patel Chest Institute, ^2^University College of Medical Sciences, University of Delhi, Delhi, India.

**Objective:**

To study the possible anti-oxidant role of nitric oxide during stress in rats using neurobehavioral and biochemical markers.

**Materials and Methods:**

Male Wistar rats (150 – 200 g, n=6 per group) were used and the study protocol was approved by the Institutional Animal Ethical Committee (IAEC). Restraint stress (RS) and cold restraint stress (CRS) were used the experimental stressors. Neurobehavioral parameters were studied by the Elevated Plus Maze (EPM) test, and gastric mucosal lesions were also assessed. Brain homogenates were analysed for NO metabolites (NOx), malondialdehyde (MDA) and reduced glutathione (GSH) by ELISA. The drugs used viz. L-arginine HCl, isosorbide dinitrate (ISDN), L-NAME and 7-NI, were administered intraperitoneally The data was analysed by using the Mann Whitney U test.

**Results:**

Restraint stress induced suppression of both open arm entries and open arm time in the EPM when compared to controls. Pretreatment with L-arginine and ISDN attenuated the RS-induced behavioral suppression, whereas, L-NAME aggravated the same. RS-induced neurobehavioral changes and its modulation by NO modulators were associated with parallel changes in brain NOx activity. RS induced enhancements in MDA and reductions in GSH levels, which were also reversed by pretreatment with NO mimetics. Further, CRS-induced gastric mucosal lesions were attenuated by the NO mimetics and aggravated differentially the NOS inhibitors. These changes were accompanied by corresponding changes in brain NOx, MDA and GSH.

**Conclusion:**

The results indicate that NO mediated anti-stress effects may be due to its ability to attenuate oxidative injury in the CNS.

**328**

**Effect of nicotine on stress-induced depression in mice**

Pachauri DR^1^, Kasture SB^1^, Kela SP^1^, Mahajan KC^1^, Khandelwal DS^2^

^1^University of Pune, India; ^2^Omnicare Clinical Research, Bangalore, India.

Stress is a physiological and behavioral response to a noxious stimulus that threatens homeostasis. Since depression is one of the most common manifestations observed in stressed animals and the frequency of nicotine self-administration among smokers increases when exposed to stressful environments, the objective of the present study was to investigate the effect of nicotine on stress-induced depression in mice. Stress was induced in mice by placing them individually for 15 min on the platform (50 cm X 6 cm) elevated to the height of 50 cm for 15 minutes. Nicotine (as bitartarate) was administered subcutaneously in the doses 0.1, 0.2 and 0.5 mg/kg prior to stress induction. Later the animals were studied for stress-induced depression using forced swimming test. Stressed animals showed significant increase in duration of immobility and decrease in latency to immobility as compared to unstressed animals. Nicotine (0.1 and 0.2 mg/kg) in stressed animals showed significant reduction in duration of immobility and increase in latency to immobility, whereas nicotine (0.5 mg/ kg) showed significant increase in duration of immobility and decrease in latency to immobility. Significant increase in duration of immobility and decrease in latency to immobility in stressed animal suggested that stress induces depression in mice. Nicotine in the dose of 0.1 and 0.2 mg/kg s.c. showed antidepressant effect, while nicotine in the dose of 0.5 mg/kg showed depressant effect in stressed animals.

**329**

**Antidepressant activity of *Sarswatarishta* in experimental models of acute and chronic depression**

Parekar RR, Dang G, Marathe PA, Rege NN

Seth G S Medical College, KEM Hospital, Mumbai, India.

**Objective:**

To evaluate *Saraswatarishta (SA)*, a polyherbal antidepressant, alone and in combination with Imipramine (Imi) and Fluoxetine (Flx) in rat models of acute and chronic depression.

**Methods:**

Animal Ethics Committee approval was obtained. In the model of acute depression, Wistar rats (n=72; 180-200 g; either sex) were randomly allocated to 2 sets of 6 groups (Group 1: DW control (5mg/kg), Group 2: Imi (30mg/kg), Group 3: Flx (20 mg/ kg), Group 4: *SA* (1.8 ml/kg), Group 5: Imi + *SA*, Group 6: Flx + *SA*) and were administered study drugs (at -24 hr, -5hr and -1hr) orally. Animals from one subset were injected reserpine (2.5 mg/kg sc; -0.5 hr). All rats were subjected to open field test (OFT) followed by forced swimming test (FST). Experimental groups for the chronic model were similar but the drugs were administered for 14 days and FST was given every day 1 hr after drug administration. One subset received reserpine on day 15 prior to OFT and FST. Motor activity during OFT and immobility time during FST were recorded in both the models.

**Results:**

*SA* shortened immobility time in the acute and chronic models, indicating a possible antidepressant activity (*P*<0.001 *vs* control). The effect was found to be comparable to Imi and Flx. Combination of *SA* with Imi and Flx showed similar results as *SA* alone. No overt behavioural changes or motor dysfunction was observed in any group.

**Conclusion:**

*SA* showed promising antidepressant activity when administered to rats in the dose of 1.8 ml/kg/day.

**330**

**Exploration of sedative and hypnotic activity of newly synthesised spirobarbitunylphenothiazines**

Yadav A, Lata S, Saxena KK, Kumar A, Srivastava VK, Goel B,

LLRM Medical College, Meerut, India.

The present study was conducted for exploring sedative and hypnotic activity of newer spirobarbitunylphenothiazines. Two such compounds (test drugs A and B), prepared in our department, were selected for the study. LD50 of these compounds was determined in albino mice of either sex. Albino rats were divided in groups (n=6) and administered the test drugs A and B in graded doses of 20, 40 and 80 mg/kg by intraperitoneal route. Sedative activity was judged by locomotor activity, exploratory activity and despair behaviour. Both the test drugs in doses 40 and 80 mg/ kg significantly inhibited locomotor and exploratory activity and significantly increased the immobility period in assessment of despair behaviour. Hypnotic activity was assessed by measuring loss of sound, pinna and righting reflexes, duration of sleep as well as effect on pentobarbitone induced sleeping time. Both the drugs in doses of 40mg/kg *per se* induced sleep similar to that induced by pentobarbitone. However, the 80mg/kg dose of both drugs produced a significant increase in the duration of sleep when compared to the pentobarbitone control. Both the drugs in doses of 40 mg/kg potentiated the pentobarbitone induced sleeping time which was comparable to chlorpromazine while in doses of 80 mg/ kg, the sleeping times surpassed that of chlorpromazine. These effects may be attributed to barbiturate moieties in the structure of the test drugs. Due to these encouraging results as for their safety and efficacy in the animal studies, these drugs appear to be potential agents for clinical studies.

**331**

**Comparative study of dual therapy in epilepsy: Efficacy and impact on quality of life**

Goyal S, Dhasmana DC, Goel D, Gupta MC, Sharma T

Pt. B. D. S. PGIMS, Rohtak; HIMS Dehradun, India.

**Objective:**

Careful evaluation of pharmacotherapy, seizure control and quality of life (QOL) are helpful in improving epilepsy care but such data are relatively meager from developing countries. This study was aimed at auditing pharmacotherapy, seizure control and QOL in persons with epilepsy who were on different combination therapies.

**Methods:**

Forty patients having generalized tonic – clonic and partial seizures were divided into four groups of ten each and were put on combinations of sodium valproate + lamotrigine (Group –I), sodium valproate + clonazepam (Group –II), oxcarbazepine + clobazam (Group-III) and phenytoin sodium + phenobarbitone (Group-IV) for a period of six months. Efficacy of various combination therapies was assessed by reduction in seizure frequency at the end of second and six months. QOL was assessed by using a 31 items Questionnaire (QOLIE-31) at zero, second and six months.

**Results:**

The various combination drug therapies used were almost equally efficacious in the treatment of epilepsy and there was no statistically significant difference amongst them. However, there was a significant improvement in QOL in phenobarbitone + phenytoin treated group as compared to valproate + lamotrigine treated group at the end of two months. However, all the treatments groups were equally effective in improving QOL at the end of six months. The adverse events were mild and tolerable with all the combination therapies.

**Conclusion:**

All the four combination drug therapies were equally efficacious in improving seizure control and QOL at the end of six month. However, larger long term prospective controlled studies are required to substantiate the findings of our study.

**332**

**Pharmacological evaluation of aqueous and alcoholic extracts of *Argyreia nervosa* boj. leaves on anxiolytic activity in mice**

Sastha Ram VK^1^, Afzal SA^1^, G Deepthi^1^, Gopi CHK^1^, Kranthi NKR^1^, Noor SM^1^ Ranganayakulu D^1^, Krishnaveni A^2^

^1^Sri Padmavathi School of Pharmacy, Tiruchanoor, Tirupathi, Andhra Pradesh, ^2^Madurai Medical College, Madurai, Tamil Nadu, India.

Anxiolytic drugs were among the most frequently prescribed drugs and as they were associated with several side effects our aim is to substitute it with traditional drugs. The objective of the present study is to evaluate the anxiolytic effect of extracts of *Argyreia nervosa* Boj. leaves in mice. Elevated plus maze (EPM), hole board test (HBT), open field test (OFT), and light dark model (LDM) were the screening tests used to assess the anxiolytic activity. Healthy male albino mice of weight 20-30g were classified into six groups each containing six animals. Diazepam (1mg/kg, i.p.) aqueous extract of leaves of *Argyreia nervosa* Boj. (100, 200mg/kg, i.p.) and alcoholic extracts of leaves of *Argyreia nervosa* Boj. (100, 200mg/ kg, i.p.) were administered 30 min before the tests. The animals receiving alcoholic extracts and diazepam showed an increase in time spent, percent entries and total entries in the open arm of EPM, increased number and duration of head poking in HBT, increased ambulation, activity at centre and total locomotion in the OFT and in LDM, the extract produced significant increase in time spent in lighted box indicating anxiolytic activity. Alcoholic extract resulted in more prominent anxiolytic activity in all the models than aqueous extracts, but was less than that produced by diazepam. Alcoholic extract of *Argyreia nervosa* Boj. leaves exhibited prominent anxiolytic activity in mice.

**333**

**Evaluation of antidepressant activity of amisulpride per se and its comparison with olanzapine and fluoxetine using forced swimming test in albino mice**

Atal S^1^, Pawar GR^1^, Agarwal RP^1^, Phadnis P^1^, Vyas S^1^, Solanki P^1^, Paliwal A^2^

^1^MGM MC Indore, M.P., India; ^2^MY Hospital, Indore, M.P., India.

**Introduction:**

Psychotic depression is a condition in which the individual manifests with primary mood symptoms and demonstrates secondary psychotic features such as delusions [fixed, false beliefs] and hallucinations [generally auditory]. Presently psychotic depression is treated along two lines of treatment i.e. combination of antipsychotic - antidepressant and electroconvulsive therapy [ECT]. Atypical antipsychotics like risperidone and olanzapine are believed to possess antidepressant properties. Substantial weight gain and sedation are their major limitations. Amisulpride is a newer antipsychotic believed to possess antidepressant property with advantages of having less incidence of weight gain and sedation.

**Materials and Methods:**

Antidepressant activity was evaluated in albino mice using both acute and chronic models of forced swimming test. Animals were divided into four groups [n=8] and subjected to the interventions as follows- Group 1- control (distilled water); Group 2- fluoxetine [10 mg/kg]; Group 3- amisulpride [70 mg/kg]; Group 4- olanzapine [2 mg/kg]. Drugs were given orally 23.5, 5 and 1 hour before the test. In acute study, drugs were given as above for one day prior to the test and in chronic study for 28 days. A pretest session of 15 min. was followed by a 5 min. test session 24 hours later. A time sampling method was used to score the behavioral activity in each group.

**Result:**

Amisulpride per se has an antidepressant activity in forced swimming test in albino mice [*P*<0.05 as compared to control]. There was no statistically significant difference between amisulpride and olanzapine. Fluoxetine showed significant antidepressant activity [*P*<0.05] when compared to amisulpride and olanzapine.

**Conclusion:**

Amisulpride per se has an antidepressant activity in forced swimming test in albino mice equivalent to that of olanzapine though less than fluoxetine.

**334**

**Differential effect of rosiglitazone in tibial and sural nerve transection and oxaliplatin induced peripheral neuropathy in rats**

Jaggi AS, Jain V, Singh N

Department of Pharmaceutical Sciences and Drug Research, Punjabi University, Patiala (Punjab), India.

The present study was designed to investigate the potential of rosiglitazone, a PPAR γ agonist; in tibial and sural nerve transection (TST) and oxaliplatin induced peripheral neuropathy in rats. Pinprick, cold immersion, acetone drop and hot plate tests were performed to assess degree of mechanical hyperalgesia, cold hyperalgesia, cold allodynia and heat hyperalgesia respectively. Myeloperoxidase assay [(MPO), a specific marker of inflammation] and calcium levels were determined to assess biochemical alterations due to neuropathy. TST and single administration of oxaliplatin (6mg/kg *i.p*.) was employed to induce neuropathy. TST resulted in development of mechanical hyperalgesia, heat hyperalgesia, cold hyperalgesia and allodynia; while oxaliplatin resulted in development of cold hyperalgesia and allodynia without altering nociceptive threshold for mechanical and heat stimuli. Administration of rosiglitazone (5 and 10 mg/kg *p.o*.) attenuated tibial and sural nerve transection induced mechanical hyperalgesia, cold hyperalgesia and allodynia except heat hyperalgesia. Further, rosiglitazone also attenuated tibial and sural nerve transection induced increase in MPO and calcium levels. However, rosiglitazone pretreatment neither modulated oxaliplatin induced alterations in pain perception nor biochemical changes. It may be concluded that rosiglitazone exerts anti-nociceptive effects in TST induced neuropathy which may be attributed to its antiinflammatory effects with subsequent decrease in calcium levels. Our results suggest that new or currently available drugs targeted at spinal PPAR γ may yield important therapeutic effects for the treatment of nerve injury induced neuropathic pain. However, PPAR γ ligands are ineffective in attenuating the state of neuropathic pain during oxaliplatin administration.

**335**

**Interactions of alprazolam and propranolol on depression, anxiety and oxidative stress**

Khanam R, Faraha A, Akhtar M, Vohora D

Faculty of Pharmacy, Jamia Hamdard, New Delhi 110062, India.

The study investigated the effects of propranolol alone and in combination with the alprazolam on depression, anxiety and oxidative stress. 48 wistar albino rats were subjected to the chronic treatment (21 days) of drugs. It was found out that propranolol alone and in combination with alprazolam exhibited antidepressant effect by significantly decreasing the immobility and increasing the swimming behavior in modified forced swim test. They also exhibited anti-anxiety effect by increasing the percentage preference, the number of entries and time spent in open arms in elevated plus maze. The combination dose was also found to decrease the oxidative stress by decreasing the malondialdehyde level and restoring the glutathione levels in rat brain. Hence, we could conclude that these combinations were found useful in the models of depression, anxiety and oxidative stress but the exact mechanism of action of these drugs is yet to be established.

**336**

**Effect of *Trapa bispinosa* on lipofuscinogenesis and fluorescence product in brain of D-galactose induced ageing accelerated mice**

Jagtap AA, Ambikar DB, Vyawahare NS, Harle UN, Virendra GK

AISSMS College of Pharmacy, Kennedy Road, Near R.T.O. Pune- 411001, Maharashtra, India.

In present investigation, effect of hydroalcoholic extract *Trapa bispinosa* (TB) was studied on lipofuscinogenesis, fluorescence product and biochemical parameter like lipid peroxidation, catalase activity and glutathione peroxidase activity in the brain of age accelerated female albino mice. The ageing was accelerated by the treatment of 0.5 ml 5% D-galactose for 15 days, which resulted in accumulation of lipofuscin granules, increased fluorescence product, increase lipid peroxidation and reduction in glutathione peroxides and catalase in cerebral cortex. The co administration of hydroalcoholic extract of TB (500 mg/kg/p.o.) with d-galactose for 15 days significantly reduced accumulation of lipofuscin granules and decreased fluorescence product in cerebral cortex. Moreover TB inhibited increase in lipid peroxidation and restored glutathione peroxidase as well as catalase activity in cerebral cortex as compare to ageing accelerated animals. To conclude 15 days administration of TB significantly prevented d galactose induced neurotoxicity. The action was mediated by its antioxidant potential.

**337**

**Effect of PARP inhibition on nerve function, nociception and oxidative stress in experimental diabetic neuropathy**

Negi G, Sharma SS

^1^National Institute of Pharmaceutical Education and Research, SAS Nagar, Mohali-160062, India.

**Introduction:**

Diabetes mellitus leads to various microvascular and macrovascular complications- the most common being neuropathy. The diversified etiologies for diabetic neuropathy include factors like metabolism, autoimmune, vascular, neuronal growth factors and oxidative stress. A fundamental mechanism in the pathogenesis suggests that free radicals and oxidants result in activation of the nuclear enzyme poly (ADP-ribose) polymerase (PARP). The data presented herein show the neuroprotective potential of Nicotinamide, a PARP inhibitor in diabetic neuropathy.

**Methods:**

Diabetes was induced using a single dose of streptozotocin (55 mg/ kg, i.p.). After six weeks of diabetes induction, animals were treated with Nicotinamide (PARP inhibitor) 100-1000 mg/kg for two weeks. Nerve blood flow, motor nerve conduction velocity and oxidative stress were assessed after two weeks of treatment. Hyperalgesia was measured using tail immersion test whereas to assess the mechanical allodynia, Electrovonfrey Anaesthesiometer was used.

**Results:**

STZ treated rats showed a significant reduction in nerve conduction velocity as compared to normal control. Also there was a decrease in nerve blood flow. The tail flick latency and paw withdrawal pressure were also significantly decreased in diabetic animals as compared to control animals. All these parameters show the development of neuropathy after six weeks of STZ administration. With two week Nicotinamide treatment, a significant (*P*<0.001) improvement in nerve blood flow was observed in treated rats as compared to diabetic rats. The decrease in tail flick latency, both in cold as well as hot immersion, was reversed significantly (*P*<0.001) as on treatment with Nicotinamide. The reduction in paw withdrawal pressure was corrected significantly (*P*<0.001) with Nicotinamide treatment.

**Discussion and Conclusions:**

Chronic treatment with Nicotinamide produced significant improvement in nerve blood flow and sensorimotor parameters. This study suggests the potential of Nicotinamide in the treatment of diabetic neuropathy.

**338**

**2-Buten-4-olide (2-B4O) mediated satiety mechanism in neonate and developing rat pups**

Mathur R

Delhi Institute of Pharmaceutical Sciences and Research, New Delhi, India.

The factors that control the satiety behavior and consequently the milk intake in neonates are complex. Mechanical stimulus of gastric filling and emptying are understood to be critical players, though not exclusive. Recently, sugar acid namely, 2-Buten-4-Olide (2-B4O) has been suggested as an endogenous satiety substance in studies conducted on adult rats and monkeys. However, the role of 2-B4O in neonate and suckling rat pups is not well elucidated and this study investigates the same. 2-B4O (50, 75 mg/kg, ip) was injected in rat pups on post natal days (0-2, 4-6, 12-14 d). A significant dose dependent inhibitory effect on milk intake was observed at all time points. In another set of experiment, 2-B4O was micro-infused into lateral hypothalamus of pups (12-14 days old). Concomitantly, it also led to a significant decrease in food intake by pups. The duration of action was a function of the 2-B4O dose and lasted up to maximum of 2h. There was no significant difference in the 24h body weight of the pups was recorded. The results of the present study implicate a critical role of 2-B4O as an endogenous satiety mediator even in neonate and suckling rat pups. The action is acute and may be mediated via central satiety mechanism(s).

**339**

**Mediation of CCK_b_ receptors in anxiety precipitated by spontaneous and naloxone-induced opiate withdrawal**

Sengaonkar VS^1^, Harle UN^1^, Gaikwad NJ^2^, Malshe JK^1^

^1^AISSMS College of Pharmacy, Kennedy Road, Pune-01, India; ^2^Department of Pharmaceutical Sciences, Nagpur University, Nagpur-440024, MS, India.

The distribution of CCK in the brain parallels that of endogenous opioidergic peptides in the various brain regions brain. CCK is reported to function via the CCK_B_ receptors and is implicated in the neurobiology of anxiety. Role of CCK in spontaneous and naloxone induced morphine withdrawal was investigated. BOC-CCK-4, the CCK_B_ receptor agonist significantly potentate and decreased onset of anxiety in both, spontaneous and naloxone-induced morphine withdrawal at the sub effective dose. However, L 365, 260 an antagonist of CCK_B_ receptor, antagonized the anxiogenic like-effect of spontaneous and naloxone induced morphine withdrawal. Locomotor activity was found to be unaffected during the spontaneous morphine withdrawal but during naloxone induced withdrawal locomotor activity was found to be increase during first 8 h. Furthermore, L365, 260 have been shown to increase the locomotor activity in naloxone induced morphine withdrawal. It may conclude that CCK receptors are involved in the anxiety during morphine withdrawal and should be further evaluated for dependence and tolerance towards the therapeutic development.

**340**

**Investigations on novel thyrotropin releasing hormone analogues on potential CNS activity**

Ingole S, Kumar S, C Meena, Monga V, Jain R, Sharma SS

National Institute of Pharmaceutical Education and Research (NIPER), Mohali, India.

**Introduction:**

Thyrotropin Releasing Hormone (TRH) is remained a choice of candidate for their potential in various CNS diseases but its short half life is of major concern. In this study we have investigated newly synthesized analogs for their CNS activity.

**Methods:**

TRH analogs were synthesized by using a multi-step synthetic procedure using solution phase peptide synthesis protocols. To study CNS activity TRH analogs were administered ten min prior to Pentobarbitone (50 mg/kg, i.p.) at the dose of 10 micromole/ kg intravenously. Sleeping time and percentage shortning was measured.

**Results:**

Five out of eighteen analogues have shortened pentobarbitone induced sleeping duration as compared to vehicle treated group, indicating their potential CNS activity. Among these analogs, NP 1841 had shown maximum shortening (68.34%) than NP 1896 (57.80%); NP 1845 (56.75%); NP 1840 (38.93%) and NP 1898 (35.24%). NP 1841 is under investigation for its potential in different disease models like memory impairment and epilepsy.

**Discussion and Conclusions:**

A TRH analog that reduces sleeping duration is considered as CNS active as it may competitively reduce the binding of PB to GABA or induce cholinergic activity as the case with TRH. Outcome of present study will indicate the discovery of CNS active TRH analog.

**341**

**Interventionary study with perindopril in experimental diabetic neuropathy**

Suruchi A^1^, Singh J^2^, Sood S^2^, Lal H^2^

^1^Dr. H S J institute of Dental Sciences Chandigarh, India; ^2^P.G.I.M.S. Rohtak, India.

Aim of the study was to evaluate role of perindopril, an angiotensin converting enzymes inhibitor in already established STZ- Diabetic Neuropathy (DN) in rats. In albino rats (200-300g) DN was produced by STZ (50mg/kg iv). MNCV was determined in sciatic posterior tibial conducting system of ether anesthetized rats by EMG. Body weight, blood/urine sugar levels, urine volume and tail flick reaction time to thermal stimulation were recorded initially, after 10 weeks of STZ and thereafter every two weeks. Rats were divided into four groups of 10 each. Group I-control Group II-STZ, Group III-STZ + perindopril (1mg/kg, po) started 10 weeks after STZ and continued for 6 weeks. Group IV − STZ + insulin (4U/kg, sc bd) started 10 weeks after STZ and continued for 6 weeks. Results of the study found that MNCV was significantly reduced after 10 weeks of STZ. However in perindopril treated group MNCV was partially improved following 6 weeks treatment. Tail flick reaction time was markedly decreased in diabetic rats (hyperalgesia), whereas perindopril treatment significantly increased the reaction time when compared to STZ diabetic rats. Though Perindopril showed no effect on body weight, it significantly improved the blood/urine sugar levels and urine volume in diabetic rats. It can be concluded that Interventionary treatment with perindopril ameliorates some neuropathic changes in STZ diabetic rats.

**342**

**Antidepressant-like effect of ethyl acetate insoluble fraction of methanolic extract of *Morus alba* l. roots in mice**

Chaudhari RS, Shimpi PC, Bhangale SP, Kanhere SV, Kawale LA, Nade VS

N.D.M.V.P.S. College of Pharmacy, Nashik, India.

**Objective:**

To evaluate the antidepressant-like effect of ethyl acetate insoluble fraction of methanolic extract of *Morus alba* L. (Moraceae) roots in mice.

**Materials and Methods:**

Forced Swim Test and Tail Suspension Test were used to study the antidepressant effect of ethyl acetate insoluble fraction of methanolic extract of *Morus alba* L. roots (25, 50 and 100 mg/kg, i.p.) in mice. Latency and duration of immobility was recorded after 30 min of treatment in both the tests. Imipramine (15 mg/kg, i.p.) and Fluoxetine (20 mg/kg, i.p.) were used as standard antidepressant drugs. Further the effect of extract was evaluated on isolated rat fundus for serotonergic effect.

**Results:**

Ethyl acetate insoluble fraction of methanolic extract of *Morus alba* significantly (*P* < 0.01) increased latency and decreased the duration of immobility in both the tests. Imipramine and Fluoxetine also significantly (*P* < 0.01) increased latency and decreased the duration of immobility. The extract significantly (*P* < 0.05) potentiated contractions produced by serotonin on isolated rat fundus.

**Conclusion:**

The results suggest that the ethyl acetate insoluble fraction of methanolic extract of *Morus alba* possesses antidepressant-like effect, which could be mediated through serotonergic mechanism. Further neurochemical and behavioural investigations can explore the mechanism of action with respect to serotonergic function and helps to establish plant as antidepressant agent.

**343**

**Anti-convulsant activity of aqueous extract of *Portulaca oleracea* leaves in albino mice**

Mayanglambam M Devi, Khomdram KP, Akham S

Regional Institute of Medical Sciences, Imphal, India.

**Introduction:**

Various species of *Portulaca* (family - Portulacacae) are used medicinally in different countries of the world such as Jamaica, North America, Indo-China etc. The leaves of *Portulaca oleracea* is claimed to cure inflammation, ulcer, urinary diseases and dysentery.

**Methods:**

Healthy albino mice (20-30 gm) were screened and divided into 5 groups of 6 animals each and received the vehicle, standard drug and the extract (200, 400, 600 mg/ kg) orally 60 mins prior to induction of convulsion. Anti-convulsant activity was tested against Maximal Electroshock (MES) by giving a current of 60 mA for 0.2 sec through transauricular electrodes. In the pentylenetetrazole (PTZ) model, the animals were grouped as in MES model and PTZ at 60mg/kg was injected subcutaneously. Abolition/reduction of Tonic hind limb extension and clonic convulsion was selected as anti-epileptic criteria in MES and PTZ model respectively.

**Results:**

The aqueous extract of *Portulaca oleracea* leaves significantly reduces the duration of tonic hind limb extension in MES. It also delays the onset and decreases the duration of clonic convulsion induced by PTZ in a dose dependent manner.

**Conclusion:**

The aqueous extract of *Portulaca oleracea* leaves was found to have significant anti-convulsant activity.

**344**

**Anti-convulsant activity of aqueous extract of *Eupatorium birmanicum* leaves**

Laishram B, Khumbongmayum S, Rajkumari B

Regional Institute of Medical Sciences, Imphal, India.

**Introduction:**

Various species of *Eupatorium* (family-Asteraceae) are used medicinally across Europe, America and Asia in various ailments as emetic, diuretic, purgative, diaphoretic, laxative and emmenagogue. The leaves of *Eupatorium birmanicum* (EB) is used traditionally in Manipur for treating various ailments like leucorrhoea, stomach ulcer and localized burning sensation on the skin.

**Methods:**

Aqueous extract (AE) of dried leaves of EB was taken using Soxhlet apparatus. Healthy albino mice of either sex weighing 20-30gms were screened and divided into 5 groups of 6 animals each and received the vehicle, standard drug and the extract (200, 400 and 600mg/kg) orally. Anti-convulsant activity was tested against Maximal Electroshock (MES) by giving a current of 60mA for 0.2sec through transauricular electrodes. Abolition or decrease in the duration of tonic hind limb extension was taken as an index of protection. In the Pentylenetetrazole (PTZ) model, the animals were grouped as in MES model. PTZ at 60mg/kg was injected subcutaneously and latency time of myoclonic spasm and clonic convulsion were noted.

**Results:**

The AE of EB significantly reduces the duration of tonic hind limb extension in MES and increases the latency of myoclonic spasm and clonic convulsion induced by PTZ (*P*<0.001).

**Conclusion:**

The aqueous extract of *Eupatorium birmanicum* significantly protects against both electroshock and chemioshock.

**345**

**Central immunoneutralization of cart modulated morphine induced anxiolytic, anorectic and antidepressant effect in rats**

Shah PP, ^1^ Harle UN, ^1^ Gaikwad NJ, ^2^Saudager AY, Kumar VK^3^

^1^Department of Pharmaceutical Sciences, Nagpur University Campus, Nagpur. ^2^Department of Pharmacology, Government Medical College, Nagpur, ^3^DBA-Biomedical Research Center, University of Delhi, Delhi, India.

Cocaine- and amphetamine-regulated transcript (CART) peptide is localized to neurons in many parts of brain including Ventral Tegmental Area (VTA), ventral pallidum, amygdala, lateral hypothalamus, and nucleus accumbens and involved in feeding, anxiety, depression and cocaine addiction. CART is closely associated with opioid receptors in many part of brain and CART is known to modulate the Dopamine, GABA and Serotonine. The interaction between CART and Opioid was evaluated for effect on anxiety, anorexia and depression. Immunoneutralization was carried out by intracerebroventricular administration of antiserum of pro-CART and six hour post-administration morphine was administered to evaluate behavioral paradigms like anxiety, anorexia and depression. Anxiety, depression and anorexia were evaluated by Elevated Plus Maze, Forced Swim Test and periodic food intake respectively. Immunoneutralization of central CART antagonized anxiolytic effect of morphine and significantly increased feeding. However, there was no change on antidepressant effect of morphine by immunoneutralization of CART. It may be concluded that CART interact with opioidergic systems to alter a behavioral response and further may play a role in opioid withdrawal and its behavioral effects.

**346**

**Intracerebroventricularly administered L-arginine impairs adaptation to stress contrary to its facilitatory influence on peripheral administration**

Ramteke DP, Shukla NR, Dixit PV, Umathe SN

Department of Pharmaceutical Sciences, Rashtrasant Tukadoji Maharaj Nagpur University, Nagpur - 440 033, Maharashtra, India.

**Objectives:**

L-arginine—a precursor for nitric oxide is reported to facilitate the adaptation to stress on peripheral administration. How does it influence the adaptation to stress on i.c.v. administration is not known, and hence addressed in the present study.

**Materials and Methods:**

Audiogenic stress was induced in mice by subjecting to broadband white noise at 100 dB intensity, produced by a white noise generator and the level of stress at different time intervals (0, 1, 3, 7, and 10 days) was assessed in terms of the anxiety, evaluated using behavioural protocols such as open field test, social interaction. The gradual change in the level of anxiety was considered as an index of adaptation. These investigations were carried out after daily administering vehicle or L-arginine via 300-mg/Kg i.p. or 10ng/mouse i.c.v. route.

**Results:**

The exposure of the mice to audio stress elevated the anxiety that gradually declined and reached to normal level on day 10 vehicle treated group. The group treated with i.p. L-arginine, exhibited low level of anxiety and its normalization on day 7 in stress exposed group. However, a group that received L-arginine via i.c.v. route continued to exhibit higher level of anxiety with no gradual decline.

**Conclusion:**

L-arginine facilitates adaptation to stress on peripheral administration, while impairs the same after i.c.v. administration, indicating some role of its metabolites in its effect on peripheral administration.

**347**

**Anticonvulsant activity of n-butanol fraction of hydro alcoholic extract of *Cestrum nocturnum* in mice**

Gangurde PR^1^, Khandare RA^1^, Vyawahare NS^1^, Harle UN^1^, Baheti JR^2^

^1^AISSMS College of Pharmacy, Kennedy Road, Near R.T.O., Pune; ^2^SSDJ College of Pharmacy, Neminagar, Chandwad, Nasik, India.

In the present investigation, we have evaluated effect of n-butanol fraction of hydro alcoholic extracts of *Cestrum nocturnum* (CNBF), against seizures induced by pentylenetetrazole (PTZ), maximal electro convulsive shock (MES) induced convulsion in mice. The different doses of CNBF 40, 80, 120 mg/kg were administered orally, sixty min later these mice were subjected to PTZ (70 mg/ kg, i.p) or maximal electroshock (45 mA- 0.2 sec) treatment. The latency to onset of convulsion was recorded in PTZ model, the duration of tonic hind limb extension (THLE) as well as lethality was recorded in MES treated mice. In PTZ model the CNBF at doses 80 mg/kg and 120 mg/kg showed significant delay in the onset of convulsion. In MES model CN 80 mg/kg and 120 mg/kg showed significant decrease in the duration of hind limb extension when compared with the vehicle treated control mice. In addition the brain GABA level was significantly increased by CNBF 80 and 120 mg/kg. Thus present study documented anticonvulsant effect of CNBF against pentylenetetrazole, maximal electroshock seizures induced convulsion in mice which maybe be due to GABAergic transmission.

**348**

**Lack of tolerance and morphine-induced cross tolerance to the analgesia of chimeric peptide of Met-enkephalin and FMRFa**

Gupta K^1^^, 2^, Vats ID^1^, Pasha S^1^, Saleem K^2^, Gupta YK^3^

^1^Institute of Genomics and Integrative Biology, Delhi, ^2^Jamia Millia Islamia, New Delhi, ^3^All India Institute of Medical Sciences, New Delhi, India.

**Introduction:**

Designed chimeric peptide of Met-enkephalin and FMRFa (YGGFMKKKFMRFa-YFa), produced naloxone reversible dose dependent analgesia and also attenuated development of tolerance to the analgesic action of morphine. In this study, YFa (80mg/kg) was tested for its tolerance and cross-tolerance behavior in relation with the standard opioid morphine (20mg/kg) on day 5 following twice daily intraperitoneal (IP) pretreatments with either YFa or morphine for 4 days.

**Methods:**

The behavioral endpoint for tolerance and cross-tolerance effect was analgesia, as seen by using a local radiant heat tail-flick analgesiometer following the opiate treatments in mice.

**Results:**

YFa did not induce tolerance and cross-tolerance effects to its analgesic action on day 5 after IP pretreatment with either YFa or morphine for 4 days. However, pretreatment of YFa (IP) for 4 days led to the development of cross-tolerance to the analgesic effects of morphine and also 4 days of pretreatment of morphine (IP) resulted in the expression of tolerance to its own analgesic effects.

**Conclusion:**

The observed effects for development of tolerance and cross-tolerance to the analgesic effect of morphine after pretreatment with either YFa or morphine might be due to the lack of reactivation of opioid receptors by morphine. On the other hand lack of tolerance and cross-tolerance to the analgesic action of YFa after pretreatment with either YFa or morphine might be due to the opioid receptors reactivation by YFa. The exact mechanism of action of YFa in terms of resistance to development of tolerance to its own analgesia and expression of cross-tolerance to the analgesic effects of morphine needs further investigation.

**349**

**Pharmacodynamic interaction of a neuroprotective herb *Acorus calamus* with conventional antiepileptic drugs in mice**

Bore VV, Yende SR, Rajgure DT, Tuse TA, Harle UN, Vyawahare NS

AISSMS College of Pharmacy, Kennedy Road, Near R.T.O., Pune, India.

*Acorus calamus* is a traditionally used as neuroprotective and anticonvulsant. Phenytoin and Phenobarbital are widely and chronically used antiepileptic drugs and known to possess side effects like cognitive impairments. The pharmacodynamic interaction between (successive methanolic) extract of *Acorus calamus* and antiepileptic like phenytoin and phenobarbital were studied using Maximal ElectroShock and Pentelenetetrazole induced seizure in mice. Administration of *Acorus calamus* at its ED_50_ dose of (185 mg/kg) significantly potentiated the anticonvulsant action of phenytoin by reducing its ED_50_ value from 13.5 mg/kg to 9.25 mg/kg; further, it potentiated the anticonvulsant action of phenobarbital by reducing its ED_50_ value from 8 mg/kg to 5 mg/kg. *Acorus calamus* showed mild to moderate anticonvulsant activity at dose of 250 mg/kg while it is ineffective at dose of (150 mg/kg) in Maximal ElectroShock and Pentelenetetrazole induced convulsion. Sub-effective dose of phenytoin as 10 (mg/kg) and phenobarbital as (2 mg/kg) when administered along with non effective dose of *Acorus calamus* (150 mg/kg), showed significant anticonvulsant activity in Maximal ElectroShock induced convulsion, while phenytoin (10 mg/kg) does not showed any significant effect in Pentelenetetrazole induced convulsion. Hence it may conclude that *Acorus calamus* reduces the dose of phenytoin and phenobarbital showed synergistic anticonvulsant action. Reduction in the doses may further contribute to decrease the side effects of antiepileptic. This study provides evidence for significance of neuroprotective herb *Acorus calamus* as an adjuvant in antiepileptic therapy for beneficial herb-drug interaction.

**350**

**Effects of panchagavya ghrita (PG) on paracetamol induced hepatotoxicity in albino rats**

Gosavi DD, Premendran J, Sachdev D

Department of Pharmacology, MGIMS, Sewagram, Wardha, (MS) India.

**Introduction:**

Sushrut Samhita mentions use of Panchagavya Ghrita (PG) in the treatment of mania, epilepsy, fever and hepatitis. In an effort to correlate the ancient knowledge with the modern concepts of research in the Pharmacology, we decided to study the effects of PG on paracetamol induced hepatotoxicity in rats.

**Materials and Methods:**

The animals were divided into four groups of 6 rats each. First two groups received PG in the dose of 1(PG1), 2 (PG2),) ml per Kg of body weight for thirty days. Third group received normal saline 2 ml per Kg orally. The fourth group acted as a standard control and received LIV 52 2ml/Kg body weight daily. Hepatotoxicity was induced with Paracetamol 1gm/Kg body weight orally once. Blood samples were collected and analyzed for liver enzymes and bilirubin. Liver was separated and estimated for Anti-oxidants (AO) in the liver tissue.

**Results:**

PG prevented the increase in the liver enzymes like AST, ALT and alkaline phosphatase produced by the paracetamol. There was no significant effect on bilirubin. It also has AO activity *in vivo* as shown by the changes in the MDA, GSH and Catalase levels.

**Conclusion:**

Hepatoprotective action of PG can be due to AO activity of PG.PG is a mixture of cow milk, ghee, urine, dung, and curd milk. Cow milk contains minerals and vitamins. Cow urine contains minerals, urea, vitamins, enzymes, and a large amount of free volatile acids with AO activity. Of these which component is responsible for AO action is difficult to comment.

**351**

**Estrogenic modulation of gait and CNS activity during hypoglycemic stress in female rats**

Verma S^1^, Srivastava RK^1^, Sood S^2^

Departments of ^1^Pharmacology and ^2^Physiology, Pt. B.D. Sharma PGIMS, Rohtak, 124001 (Haryana), India.

**Objective:**

Ovarian steroid hormones play an important role in the regulation of carbohydrate metabolism in the brain and estrogen has subtle modulatory effect on brain glucose uptake. The present study was taken up to assess the role of estrogen on hypoglycemia induced changes in gait and CNS activity in female rats.

**Methods:**

Insulin (2U/kg, IP) induced hypoglycemia was produced in ovariectomized adult female albino rats which received chronic treatment with (OVX+EB) estradiol benzoate (100*µ*gm/kg, IP) or vehicle (OVX+Veh) for 2 weeks. Changes in gait and CNS activity were assessed using Y-shaped runway and Open-field test respectively before and after insulin-hypoglycemia.

**Results:**

The gait of rats showed similar width or splay in different pre-treatment groups before hypoglycemia. However, significant increase (f_(3, 33)_=4.01, *P*<0.01) in splay and log variance was seen in OVX+EB rats indicating marked increase in ataxia during hypoglycemia. Significant decrease in CNS activity, with decrease in ambulation, rearing, grooming and mobilization was observed in estradiol treated ovariectomized rats during hypoglycemia.

**Conclusion:**

Estradiol benzoate further aggravated the ataxia and CNS depression during insulin hypoglycemia in female rats.

**352**

**Possible neurobehavioral, biochemical and neurochemical mechanisms in sleep-deprivation induced memory dysfunction in rat**

Kalonia H, Bishnoi M, Kumar A

Punjab University, Chandigarh, India.

**Introduction:**

Frequent sleep deprivation disrupts various neurobehavioral and neurochemicals necessary for homeostasis of a human being. The present study was designed to investigate the possible mechanisms of sleep-deprivation-induced memory dysfunction by using different behavioral, biochemical and neurochemical parameters.

**Materials and Methods:**

Male Wistar rats were sleep deprived for 72 h using a grid suspended over water. Elevated plus maze, Passive avoidance and Morris water maze tests were used to assess memory retention in 72-h sleep-deprived animals. Various electrophysiological parameters (sleep–wake cycle), biochemical (lipid peroxidation, reduced glutathione, nitrite, catalase, acetylcholinesterase enzyme) and neurochemical parameters (norepinepherine, dopamine and serotonin) were also assessed.

**Results:**

The 72-h sleep deprivation caused memory dysfunctions in all the behavioral paradigms, alteration in sleep–wake cycle (delayed sleep latency, shortening of non-rapid eye movement [non-REM] and rapid eye movement [REM] sleep and increased waking period), oxidative stress (increased lipid peroxidation and nitrite levels, depletion of reduced glutathione and catalase activity). Besides, there were increased levels of acetylcholinesterase (the enzyme causing the degradation of acetylcholine) and reduction in norepinephrine and dopamine levels in 72-h sleep-deprived animals.

**Conclusion:**

The 72-h sleep-deprivation-induced memory deficit possibly could be due to combined effect of oxidative damage as well as alteration in neurotransmitter levels.

**353**

**Effect of chyawanprash in the prevention of dementia**

Bansal N^1^^, 2^, Parle M^2^

^1^Rajendra Institute of Technology and Sciences, Sirsa, ^2^Guru Jambheshwar University, Hisar, India.

The present study was aimed at investigating the effects of Chyawanprash on memory, brain cholinesterase activity and brain thiobarbituric acid reactive substances (TBARS) in mice. Chyawanprash is an Ayurvedic formulation, which includes more than 50 herbs. In Ayurveda, Chyawanprash is classified under the category of Rasayana, which aims at maintaining physique, vigor and vitality, while delaying ageing process. Chyawanprash was administered orally in two doses (50 and 100 mg/Kg) for fifteen successive days in different groups of mice. Moris water maze apparatus served as exteroceptive behavioral model. Scopolamine (1.4 mg/Kg, i.p) and alprazolam (0.5 mg/Kg) induced amnesia served as interoceptive behavioral models. Brain cholinesterase activity and brain TBARS levels also estimated. Chyawanprash (50 and 100 mg/Kg, p.o.) produced a dose-dependent prevention in memory deficit (induced by scopolamine and alprazolam) in mice as indicated by the improved memory scores using Moris water maze apparatus in comparison to scopolamine as well as alprazolam control groups. Furthermore, administration of Chyawanprash also prevented increase in brain cholinesterase activity and brain TBARS levels. Chyawanprash may prove to be a useful remedy for the management of dementia due to its prevention in memory deficit, anticholinesterase activity and antioxidant activity.

**354**

**Celecoxib, a selective cyclooxygenase-2 (COX-2) inhibitor ameliorates perphenazine-induced catatonia in rats**

Gupta A, Dhir A, Kumar A, Kulkarni SK

Panjab University, Chandigarh, India.

**Introduction:**

Cyclooxygenase (COX) is the rate-limiting enzyme involved in the formation of prostaglandins and has been speculated to play an important role in the pathophysiology of Parkinson’s disease. However, the exact mechanism of antiparkinsonian effects of COX-inhibitors is not known. With this background, the present study aimed to explore the protective effect of celecoxib (a selective COX-2 inhibitor) if any, against perphenazine-induced catatonia using bar test. The study was further extended to assess the extent of oxidative damage in rat’s brain following perphenazine and its modulation by celecoxib.

**Materials and Methods:**

Male wistar rats (150-200 gm), bred in Central Animal House (CAH) of Panjab University, Chandigarh were used. All animal protocols were approved by the Institutional Animal Ethics Committee (IAEC). Bar test was employed to assess the severity of catatonia after perphenazine administration.

**Results and Discussion:**

Administration of perphenazine (5 mg/kg, i.p.) increased the time spent on 9 cm high bar, the maximum response reached at 4 hours. Perphenazine administration significantly increased lipid peroxidation, brain nitrite concentration, depleted reduced glutathione and superoxide dismutase levels. Pretreatment of celecoxib (10-40 mg/kg, p.o) decreased the severity of perphenazineinduced catatonia and oxidative damage. Furthermore, celecoxib at sub-effective dose (10 mg/kg, p.o.) potentiated the effect of lower dose of combination of L-DOPA (50 mg/kg, p.o) and carbidopa (10 mg/kg, p.o.).

**Conclusion:**

Celecoxib attenuated the perphenazine-induced catatonia by modulating the dopaminergic neurotransmission and/or oxidative stress. COX-2 inhibitors may have a role as adjunct drug therapy in Parkinson’s disease.

**355**

**Protective effect of alpha lipoic acid on cognition derangement induced by topiramate in pentylenetertrazole (PTZ) kindled mice**

Bharal N, Mediratta PK, Sharma KK

University College of Medical Sciences and GTB Hospital, Delhi-110095, India.

**Introduction:**

Cognitive problems are encountered by more than half of epileptic patients receiving antiepileptic drug (AED) treatment. Thus there is a need of a novel add-on drug which could prevent cognitive impairment associated with AED treatment. The present study aimed to investigate the effect of alpha lipoic acid on cognitive derangement produced by PTZ-kindling and topiramate treatment during kindling.

**Methods:**

Kindling was induced in Swiss albino mice by injecting PTZ (25 mg/kg, i.p.) once every 2 days for 5 weeks. Alpha lipoic acid (100 mg/kg, p.o.) and topiramate (10mg/kg, p.o.) were administered alone and in PTZ treated animal till 5 weeks. At the end of 5^th^ week cognition tests were performed on elevated plus maze to assess transfer latency and passive avoidance test to assess step down latency. After behavioral experiment MDA (malondialdehyde) and reduced glutathione levels were estimated, as markers of oxidative stress, in whole brain tissue of mice.

**Results:**

Topiramate treatment has significantly impaired cognition in PTZ-kindled animals. However, administration of alpha lipoic acid along with topiramate in PTZkindled group has significantly improved memory as evidenced by decreased transfer latency and increased step down latency. Further alpha lipoic acid treatment has also decreased oxidative stress as evidenced by decreased MDA and increased reduced glutathione levels in brain tissues of both PTZ-kindled and topiramate treated- PTZ-kindled mice.

**Conclusion:**

It can be concluded that alpha lipoic acid improve cognition derangement induced by topiramate in PTZ-kindled mice.

**356**

**Caffeic acid prevents colchicine induced cognitive dysfunction and oxidative stress in rats**

Mehan S, Bansal V, Goyal A, Sharma V, Deshmukh R, Bedi KL

I.S. F. College of Pharmacy, Moga, Punjab-142001, India.

**Introduction:**

To evaluate the neuroprotective efficacy of Caffeic acid in cognitive dysfunction and oxidative stress induced by intracerebroventricular (ICV) colchicine in rats.

**Materials and Methods:**

Colchicine (COL) (15*µ*g/5*µ*l) was given intracerebroventrically (ICV) to induce experimental dementia of Alzheimer’s type in male wistar rats (260-300g). Rats were treated chronically with caffeic acid (10, 20 and40 mg/kg) for a period of 21 days. Learning and memory was assessed with Morris water maze and elevated plus maze. Locomotor activity was examined with photoactometer and brain acetylcholinestrase activity was measured by Ellman method. Malonialdehyde, glutathione and nitrite levels were measured by Wills *et al*., Ellman *et al*., and Green *et al*., respectively. Lactate dehydrogenase (LDH) was estimated with commercially available kit and the total protein content was measured by Biuret method.

**Results:**

ICV COL treated rats exhibited poor retention of memory in Morris water maze and modified elevated plus maze task paradigms. Chronic treatment with caffeic acid dose dependently improved learning andmemory and significantly reduced elevated levels of malondialdehyde, nitrite and restored depleted levels of glutathione. It decreased elevated levels of AChE and LDH confirming its effect on cholinergic transmission and cell viability.

**Conclusion:**

ICV COL in rats mimic changes observed in Alzheimer kind of dementia i.e impaired cholinergic neurotransmission, oxidativenitrosative and subsequent neuronal death. Results of present study confirms candidature of caffeic acid as a possible drug target in finding a cure for Alzheimer’s disease.

**357**

**Caffeic acid reverses cognitive dysfunction and oxidative stress inducedc by intracerebroventricular administration of colchicine**

Singh J, Bansal V, Sharma V, Deshmukh R, Bedi KL

I.S. F. College of Pharmacy, Moga, Punjab-142001, India.

**Objective:**

To evaluate the neuroprotective efficacy of Caffeic acid in cognitive dysfunction and oxidative stress induced by intracerebroventricular colchicine in rats.

**Materials and Methods:**

Male wistar rats (250-280g) were used in the study and were given Colchocine intracerebroventrically(15*µ*g/5*µ*l) to induce experimental dementia of Alzheimer’s type. The rats were treated chronically with Caffeic acid (10, 20 and40 mg/kg) for a period of 21 days starting soon after colchicine administration. Learning and memory was assessed with Morris water maze and modified elevated plus maze task paradigms. Locomotor activity was examined with photoactometer. Brain acetylcholinestrase and nitrite levels were measured by Ellman method., malonialdehyde, nitrite and glutathione activity was measured by Wills method, Ellman *et al.*, and Green *et al.*, respectively. Lactate dehydrogenase (LDH) was estimated with commercially available kit and the total protein content was measured by Biuret method

**358**

**Dual inhibition of COX and LOX reverses cognitive dysfunction and oxidative stress inducedc by intracerebroventricular administration of streptozotocin**

Singh P, Sharma V, Deshmukh R, Bedi KL

I.S.F. College of Pharmacy, Moga, Punjab-142001.

**Objective:**

To evaluate the neuroprotective efficacy of inhibitors of cyclooxygenase and lipoxygenase in cognitive dysfunction and oxidative stress induced by intracerebroventricular streptozotocin in rats.

**Materials and Methods:**

Present study was conducted on male wistar rats (250-280g) and they were given streptozotocin intracerebroventrically (3mg/kg) on days 1 and 3 to induce experimental dementia of Alzheimer’s type. The rats were divided into different groups and were treated chronically with Nimuselide(COX inhibitor), Caffeic acid(LOX inhibitor) and combination of both i.e caffeic acid +nimuselide for a period of 21 days starting from 1^st^ day of streptozotocin administration in different doses. Learning and memory was assessed by Morris water maze. Locomotor activity was examined using photoactometer. Brain acetylcholinestrase and glutathione levels were measured by Ellman method. Malonaldehyde and nitrite activity was measured by methods described by Wills and Green respectively. Lactate dehydrogenase was estimated with commercially available kit.

**Results:**

ICV STZ treated rats exhibited poor retention of memory in Morris water maze and increased oxidative-nitrosative stress, cholinergic dysfunction and cell death. Although chronic treatment with nimuselide, Caffeic and their combination dose dependently reversed all the changes induced by ICV STZ but combination of drugs used was found to be more effective.

**Conclusion:**

Present study support the dual concept of inhibition of cyclooxygenase and lipooxygenase as dual inhibtion was found to be more effective and it also predicts involvement of both isoforms in neurocognitive deficits associated with dementia resembling that of Alzheimer type.

**359**

**Study of combined effect of calcium channel channel blockers with anti-epileptic drugs in maximum electroshock and pentylenetetrazol induced convulsions**

Brahmane R, Dahat S

Mahatma Gandhi Institute of Medical sciences, Sewagram- Wardha, India.

**Introduction:**

Present antiepileptic drugs unable to control seizures effectively. Limitations highlighted need for developing newer agents for epilepsy.

**Materials and Methods:**

Effect of phenytoin sodium 15 mg/kg, sodium valproate 300 mg/kg and carbamazepine 8 mg/kg alone and in combination with cinnarizine 30 mg/kg, nimodipine 21 mg/kg and nifedipine 5 mg/kg studied in albino mice i.e 12 mice in each group. Seizures were induced by MES by using electroconvulsiometer and by pentylenetetrazol (PTZ) induced seizures. Abolition of hindlimb tonic extension was an index of anticonvulsant activity in MES. Failure to observe single episode of tonic spasms for 5 second duration for one hour was index of protection in PTZ seizures. With this percentage protection calculated. Combined drugs are compared with antiepileptic drug alone to which they are combined. For analysis formula of critical ratio applied.

**Results:**

In MES seizures augmented effects obtained when cinnarizine and nifedipine added to phenytoin sodium i.e 66.66%; nimodipine added to carbamazepine i.e 66.66%; cinnarizine and nimodipine are combined with sodium valproate i.e 100%. In PTZ induced seizures augmented effects obtained when nimodipine combined with phenytoin sodium i.e 66.66%, cinnarazine and nifedipine combined with carbamazepine i.e.66.66%, nifedipine and nimodipine combined with sodium valproate i.e.100%.

**Dicussion and Conclusion:**

Cinnarazine given concurrently with sodium valproate produces significant protection against MES seizures. Nimodipine along with sodium valproate produces significant protection against both MES and PTZ induced seizures. Nifedipine along with sodium valproate produces significant protection against PTZ induced seizures. The results provides potential benefit of combining calcium channel blockers with sodium valproate in refractory epilepsy.

**360**

**Allopregnanolone attenuates nicotine withdrawal anxiety and restores locomotor activity in mice**

Panpaliya VB, Tundulwar MR, Chopde CT

S.K.B college of pharmacy, Nagpur, India.

**Introduction:**

Termination of nicotine administration shows withdrawal symptoms, characterised by anxiety, irritability, and decrease in l activity. Allopregnanolone a metabolite of progesterone is positive modulator of GABA_A_ receptor complex and exhibits anxiolytic effect. It has also been found to prevent the dependence to ethanol and morphine and reduced ethanol withdrawal anxiety.

**Methods:**

Nicotine dependence was produced in male swiss albino mice by nicotine injections (2 mg/kg, s.c, four times a day) for 10 days. Mice were withdrawal on 11^th^ day. Allopregnanolone treatment(1 mg/kg, i.p) was given either daily on 8^th^, 9^th^ and 10^th^ day or during withdrawal period on 11^th^ day), and withdrawal symptoms were evaluated at 30 min, 4h, 8h, 12h and 24h post nicotine withdrawal in elevated plus maze and actophotometer for anxiety and locomotor activity respectively.

**Results:**

Nicotine withdrawal might exhibit time dependent increase in anxiety and peak activity seen at 24 hour. Whereas, locomotor activity was significantly decreased during nicotine withdrawal. Allopregnanolone administration during both the phases i.e. nicotine dependence phase (8^th^, 9^th^ and 10^th^ day) and withdrawal phase (11^th^ day) significant inhibited withdrawal anxiety by increasing percent open arm entry and time spent in open arm. It also reversed the nicotine withdrawal induced decrease in locomotor activity.

**Conclusion:**

Allopregnanolone administration either during development phase or withdrawal phase attenuates nicotine withdrawal symptoms. Thus present study highlights the importance of neurosteroid allopregnanolone in nicotine dependence and addiction. It may help to develop further strategies to treat the nicotine addiction.

**361**

**Chronic treatment of *Withania somnifera* prevents ethanol withdrawal symptoms in rats**

Wankhade PW, Kedia AS, Kotagale NR, Chopde CT

S. K. B College of Pharmacy, Nagpur, India.

**Introduction:**

Alcoholism is a chronic relapsing disorder, accompanied by alterations in psychological and physiological functioning which reaches to an addictive state. Abrupt cessation of prolonged alcohol consumption causes withdrawal syndrome such as tremors, muscular rigidity, delirium, anxiety, depression, hyperlocomotion and seizures. Alcohol withdrawal is also associated with hypercortisolaemia and increase in salivary cortisol levels. Whereas, *Withania somnifera* exhibits anti-inflammatory, antistress, immunomodulatory, adaptogenic activity etc. Antalarmin CRF_1_ receptor antagonist which are directly activated in alcohol withdrawal.

**Methods:**

Ethanol dependence was induced in male Sprague Dawley (200-250g) rats by chronic exposure to alcohol containing (7.2%v/v) liquid diet for 21 days. Chronic alcohol treatment was discontinued on 22^nd^ day. In separate groups *Withania somnifera* (25 or 50 mg/kg, i.p.) was administered during development phase (from day 1-21) or from day 15–21 of alcohol dependence. Alcohol withdrawal anxiety and locomotor activity were measured at 24 h post alcohol withdrawal and biochemical estimation of blood glucose, cortisol and malondialdehyde was carried out.

**Results:**

The chronic (21 days) and subchronic (7 days) treatment with *Withania somnifera* (25 or 50 mg/kg, i.p.) and combination of *Withania somnifera* (25mg/kg) with antalarmin (5 mg/kg, i.p.) significantly (*P* < 0.001) prevented the ethanol withdrawal symptoms such as anxiety and locomotor activity and reduced the glucose, cortisol and malondialdehyde levels in ethanol dependant rats.

**Conclusion:**

Present study demonstrated that *Withania somnifera* reduces alcohol withdrawal symptoms such as increased anxiety and locomotor activity. It is possible that adaptogens including *Withania somnifera* may be helpful in preventing alcohol dependence and relapse.

**362**

**Current status of herbal drugs in Alzheimer’s disease**

Goyal A, Sharma V, Deshmulh R, Bedi KL

Deptt. of Pharmacology, ISF College of Pharmacy, Moga-Punjab, India.

Alzheimer’s disease is a most common, age related cognitive syndrome that is characterized behaviorally by learning and memory impairment, abstract thinking, loss of mathematical skills, aggression, anxiety and convulsions in later stages. Pathologically it is characterized by amyloid ß, neurofibrillary tangles, hippocampal atrophy, synapse and neuronal loss. At present there are acetylcholinestrase inhibitors (Tacrine, Donepezil, Galantamine and Rivastigmine) and NMDA antagonist (Memantine) as approved drugs for treatment of Alzheimer’s disease. Unfortunately they are neither able to stop the cascade of disease nor are free from side effects. On the other hand some herbal drugs like Ginkgo biloba, Ginseng, vitamin E, *Withania somnifera, Eclipta elba, Ginger oficianlis, Bacopa monera, Centella asiatica, Herbacium gossypium, Acorus calamus, Cynodon dactylon* and vinpocetne have shown exciting outcomes in this area. These agents have shown not only antistress, adaptogenic and antioxidant effect but they have also shown memory enhancing effect as well. Thus beside development of synthetic drugs equal attention must be devoted to standardization, characterization and development of herbal drugs.

**363**

**Evidence for the role of nitric oxide in adaptogenic effects of some centrally acting drugs**

Rashmi Anand, Gulati Kavita, Ray Arunabha

Department of Pharmacology, VallabhBhai Patel Chest Institute, University of Delhi, Delhi - 110007, India.

**Objective:**

The present study evaluated the possible role of NO in the adaptogenic effects of two centrally acting drugs, diazepam and morphine, in rats.

**Materials and Methods:**

Inbred male Wistar rats (150 – 200g) were used and the protocol was approved by the Institutional Animal Ethical Committee. Restraint stress (RS) was used as experimental stressor and neurobehavioral parameters in elevated plus maze (EPM) and biochemical markers (brain NOx) were assessed, after various pre-treatments. The effects of diazepam and morphine were compared with vehicle controls, and the results were analysed using the Mann-Whitney U test.

**Results:**

RS exposure reduced open arm entries (OAE) and time (OAT) by 75% and 80%, respectively, from the values of the control (no RS) group, in the EPM. These RS-induced neurobehavioral changes were associated with significant suppression in NOx activity in brain homogenates, which was reduced by 50% as compared to control values. Diazepam (0.5 and 1mg/kg) attenuated RS-induced neurobehavioral activity in EPM and also induced elevations in brain NOx activity, in a dose related manner. Pretreament with NO precursor, L-arginine, potentiated diazepam effects on the EPM, whereas, the NOS inhibitor, L-NAME attenuated the same. Similar changes in EPM activity and brain NOx levels were seen after pretreatment with morphine (2.5 and 5mg/kg) alone and in combination with NO modulators.

**Conclusion:**

These results suggest the possible involvement of NO in the observed ant-stress / adaptogenic effects of both diazepam and morphine, and highlight its importance as a crucial regulator of stress responses.

**364**

**The role of histone deacetylases (HDAC)-induced brain chromatin remodeling in rapid alcohol tolerance**

Sakharkar A J, Tang L, Zhang H, Pandey S C

University of Illinois at Chicago, and Jesse Brown VA Medical Center, Chicago, USA.

Histone deacetylases (HDACs) have emerged as potential therapeutic targets for several CNS disorders. Recently, we have shown that trichostatin A (TSA), an HDAC inhibitor, can prevent alcohol withdrawal-related anxiety, while restoring histone acetylation levels and neuropeptide Y (NPY) expression in the amygdala of rats. However, the role of chromatin remodeling in the development of rapid tolerance to the anxiolytic effects of alcohol is less clear. Here, we observed that a single exposure [saline on the first day and ethanol (1g/kg) on the second day] to ethanol produced an anxiolytic response, inhibited HDAC activity, and increased histone acetylation and NPY expression in the amygdala of rats. On the other hand, two exposures to ethanol (1g/kg each), 24 hours apart, did not produce an anxiolytic response after the second exposure to ethanol, as measured by the light dark box exploration (LDB) and elevated plus maze (EPM) tests. In addition, HDAC activity, histone acetylation, and NPY expression in the amygdala was unaffected in alcohol tolerant rats compared to control rats. Interestingly, acute TSA treatment prevented the development of tolerance to the anxiolytic effects of ethanol. TSA also inhibited HDAC activity, and increased histone acetylation and NPY expression in the amygdala of alcohol tolerant rats. These results suggest that HDACs induced changes in histone acetylation and NPY expression may be involved in the development of rapid tolerance to the anxiolytic effects of ethanol (Supported by the grants from NIAAA-NIH and VA merit and Career Scientist Award Grant to SCP).

**365**

**Assessment of efficacy and safety of levetiracetam as add on therapy in poorly controlled epileptic patients**

Gupta PP, Mishra P, Thacker AK^1^, Pandey ON^2^

Dept. of Pharmacology; Neurology^1^; S.P.M.^2^; B.R.D.Medical College, Gorakhpur; (U.P.), India.

**Introduction:**

Despite availability of more than 20 Conventional and Newer Antiepileptic drugs, seizures are not adequately controlled in about 30% patients. In the present study Levetiracetam, a new Antiepileptic drug having different preclinical profile as well as several novel mechanisms of action, was evaluated for its efficacy and safety as add on therapy in patients with poorly controlled / refractory epilepsy

**Methods:**

In this, noncomparative, open label single centre study after following inclusion criteria 77 patients were given Levetiracetam for a total of 24-28 weeks which included initial base line period of 12 weeks on their current Antiepileptic Drugs followed by addition of Levetiracetam – titration phase of 4-8 weeks, thereafter remaining period of maintenance phase. Levetiracetam was given in the maximum effective / tolerated dose starting with 250- 500 mg daily with increments of 250 to 500 mg daily every two weeks depending on clinical response, adverse effects and age of patients. Efficacy was measured by primary and secondary end points. The safety was assessed by physical, neurological examination and laboratory investigations.

**Results:**

Primary end point of more than or equal to 50% reduction in seizure frequency was observed in 73.6% patients (responders) while 26.4% could not achieve this primary end point(non-responders).Statistically the difference between responders and non-responders was found to be highly significant. In terms of secondary end points i.e.mean seizure frequency reduction, percentage seizure day count reduction and seizure duration reduction showed a highly significant difference between responders and non-responder patients. 20.8% achieved seizure freedom which belonged to partial and generalized categories while none of multiple seizures patients became seizure free. Highest responder/non-responder ratio was observed for max. dose of 1000 mg/day of Levetiracetam followed by 750 mg/day with ratio of 9:1. Surprisingly in 9 patients seizure frequency increased on dose escalation above 1000 mg/ day. 50% have no ADR and rest experienced tolerable one or two ADR pertaining to CNS or GIT. Notable ADR in the form of exacervation of seizure frequency was seen in 12.5% patients who belonged to complex partial seizure. Overall 9% patients developed severe ADR leading to discontinuation.

**Conclusion:**

Study revealed that Levetiracetam is a useful drug as add on therapy in all types of seizure except multiple seizure. The most effective dose was 1000 mg/day. Drug related ADR and in some patients exacervation of seizures alongwith cost of the drug are the limiting factors in Levetiracetam addition.

**366**

**Nootropic activity of thiazolidinediones**

Kumar Neeraj, Srivastava DN, Jain AK, Katewa Jitesh

B.R. Nahata college of Pharmacy, Mandsaur, India.

**Introduction:**

Memory is a complex function of the brain that uses several storage buffers of differing capacity and duration. It can be divided into three major types: working, episodic, and long-term, or remote, memory. Memory is the most common cognitive ability lost with dementia; 10% of persons over age 70 and 20 to 40% of individuals over age 85 have clinically identifiable memory loss. This study evaluates Thiazolidinediones as memory enhancers on scopolamine induced albino rat model using Cook’s Pole Climbing Response Apparatus.

**Methods:**

The Nootropic activity of Thiazolidinediones was evaluated by using the Conditioned Avoidance Response (Through Cook’s Pole Climbing Response Apparatus) in rats as described by Cook and Weidley (1957). Rosiglitazone (2 and 5 mg/kg p.o.) and Pioglitazone (10 and 20 mg/ kg p.o.) were taken as test drugs. Piracetam (100 mg/kg p.o.) was taken as standard drug for memory enhancement. Scopolamine butylbromide (1 mg/kg i.p.) was used to induce amnesia in albino rars. Albino rats will be divided into seven groups of six animals in each group. Daily training was given to albino rats till they achieve 95-99% avoidance.

**Results:**

Reduction of number of days to achieve 95% Avoidance, indicating improvement of memory and successfully reversed the amnesia induced by scopolamine. No. of days are less in case of Thiazolidinediones and statistically significant.

**Conclusion:**

This study, Evaluation of Thiazolidinediones on memory models concluded that Thiazolidinediones successfully reversed the amnesia in scopolamine induced amnesia albino rat models using Cook’s Pole Climbing Response Apparatus means Thiazolidinediones have Nootropic potenrial.

**367**

**Study of antipsychotic activity of newly synthesized spirobarbitunylphenothiazines**

Lata S, Saxena KK, Kumar A, Bhasin N, Srivastava VK, Goel B

LLRM Medical College, Meerut, India.

The present study was conducted to ascertain the antipsychotic activity of newly synthesized spirobarbitunylphenothiazines. Two such compounds (test drugs A and B), synthesized in out department were selected for the study. LD50 of these compounds were determined on albino mice of either sex. Albino rats of either sex were divided into groups (n=6) and administered the test drugs A and B in graded doses of 20, 40 and 80mg/ kg by intraperitoneal route. Induction of catalepsy, reversal of The present study was conducted to ascertain the antipsychotic activity of newly synthesized spirobarbitunylphenothiazines. Two such compounds (test drugs A and B), synthesized in out department were selected for the study. LD50 of these compounds were determined on albino mice of either sex. Albino rats of either sex were divided into groups (n=6) and administered the test drugs A and B in graded doses of 20, 40 and 80mg/ kg by intraperitoneal route. Induction of catalepsy, reversal of amphetamine/apomorphine induced stereotyped behaviour and tranquilization were taken as indicators of antipsychotic activity. At doses of 40 and 80mg/kg, both drugs induced catatonia which was comparable to haloperidol induced catatonia. Pretreatment with either of the test drugs in doses of 80mg/kg significantly prevented the induction of stereotyped behaviour by amphetamine or apomorhine similar to the effect of chlorpromazine taken as control. Tranquilization was studied by observing the effect of the test drugs on motor coordination (Rotarod performance test) and avoidance of conditioned reflex (pole climbing method). Both the test drugs in doses of 40mg/kg displayed motor in-coordination which was equivalent to that shown by chlorpromazine control. However, in 80mg/kg doses, motor in-coordination was marked and even superseded the effect of chlorpromazine. Both the test drugs, despite exhibiting some effect, could not match the efficacy of chlorpromazine in producing avoidance of conditioned reflex at any of the administered doses. The observed effects were expected, in view of the fact that the test drugs are, in fact, phenothiazines. The encouraging results delivered by the test drugs, rightfully gives them the status of potential candidates for clinical studies.

**368**

**Role of steroids and mitochondrial oxidative stress in the progression of secondary injury induced by neonatal asphyxia**

Reddy NR^1^, Sairam K^1^

^1^Banaras Hindu University, Varanasi, India.

**Introduction:**

Neonatal Asphyxia (NA) accounts for 16% of total neonatal mortality. It is due to combination of hypoxia and ischemia in neonates, occurring around delivery or birth. The acute phase of asphyxia leads to hypoxic injury in brain especially, as it is dependent on aerobic respiration. Reestablishment of normal respiration after short phase of hypoxia aggravates progression of secondary injury, from 80 hours to 8 days of acute injury. The infant who survives this hypoxic injury is prone to long-term abnormalities like mental retardation, cerebral palsy, epilepsy etc. Generation of free radicals from mitochondrial swelling and dysfunction is considered to be on of the main causes for secondary injury. Though its pathophysiology in cause of this secondary injury is established, impact of steroids on mitochondrial oxidative stress is not well defined. The present study is designed to study this relationship as a target to limit secondary damage from NA.

**Methods:**

Ischemic hypoxia model: Unilateral carotid artery occlusion was induced in rat pups of 4-7 days old, which were then immediately exposed to 8% O_2_ + 92% N_2_ for 2 hrs. Then the occlusion was removed and the animals were allowed to recapitulate until further experimentation.

**Results and Conclusion:**

A significant influence of steroid on mitochondrial oxidative stress was observed. This pathway may be potentially manipulated to limit secondary injury due to NA.

**369**

**5-HT3 receptor antagonist blocks the expression of ethanol induced behavioural sensitization**

Raut VS, Bhutada PS, Umathe SN

Department of Pharmaceutical Sciences, Rashtrasant Tukadoji Maharaj Nagpur University, Nagpur - 440 033, Maharashtra, India.

**Objective:**

Ondansetron (5-HT3 receptor antagonist) is known to attenuate cocaine and methamphetamine induced behavioural sensitization. Several reports indicate serotonergic involvement in ethanol withdrawal behaviour. The present study investigates whether it can be also useful against behavioural sensitization in ethanol withdrawal state in mice.

**Materials and Methods:**

Behavioural sensitization evident in alcohol addicts is simulated in mice by repeatedly administering ethanol (2.4 g/kg, i.p.), on day 1, 3, 7, 10 and then administering challenge dose on day 15 that leads to exceptionally higher locomotor activity and considered as a marker of behavioural sensitization. Locomotor activity test was assessed by actophotometer. The studies were carried out in groups that received ondansetron (0.25-1.0 mg/kg, s.c.) 30 min prior to challenge dose of ethanol; and in another group, prior to repeat ethanol except that of the challenge dose. The blood EtOH concentration was determined by UV spectrophotometry.

**Results:**

The results indicate that ondansetron blocked the expression of acute ethanol-induced locomotor sensitization but did not influence the development of sensitization to locomotor stimulant effect of ethanol. The data collectively indicates that the effect of ondansetron on expression of ethanol induced behavioral sensitization is not due any change in blood ethanol levels or any per se sedative effects.

**Conclusion:**

The results suggest the utility of 5-HT3 receptor antagonist to prevent the ethanol-induced behavioral sensitization.

